# Dynamic F1-score-based voting strategies for multi-class classification: an adaptive ensemble approach for non-linear and imbalanced datasets

**DOI:** 10.1038/s41598-026-67363-7

**Published:** 2026-08-25

**Authors:** Amr M. Hamed, Abdel-Fattah Attia, Heba El-Behery

**Affiliations:** Department of Computer Engineering and Systems, Faculty of Engineering, Kafrelsheikh University, Kafrelsheikh, Egypt

**Keywords:** Ensemble Learning, Dynamic Voting, Multi-Class Classification, Medical Diagnosis, F1-Score-Based Weighting, Soft Voting, Breast Cancer Detection, Heart Disease Prediction, Cancer, Computational biology and bioinformatics, Mathematics and computing

## Abstract

Classification is a core machine learning task, and ensemble voting methods are widely used to improve predictive accuracy in domains such as medical diagnosis, where class imbalance and non-linear decision boundaries are common. Conventional strategies: Majority Voting (MV), Weighted Voting (WV), and Soft Voting (SV) rely on static or classifier-level weighting schemes that fail to capture per-class differences in classifier reliability. Three dynamic, class-specific voting strategies are introduced: Highest Class F1-Score Voting (HCF1V), Cumulative Class F1-Score Voting (CCF1V), and Enhanced Class F1-Score Voting (ECF1V), each assigning classifier weights based on per-class F1-scores obtained during validation rather than overall performance. The strategies were evaluated through computational simulation on three synthetic non-linear datasets (Gaussian Mixture, Spiral, and Moon) and two real-world medical benchmarks—the Breast Cancer Wisconsin Dataset (BCWD) and the UCI Heart Disease Dataset (UHDD)—using scikit-learn–based classifiers, with statistical significance assessed via Wilcoxon signed-rank tests. ECF1V achieved the highest accuracy across most settings, reaching 98.25% on BCWD and 89.47% on UHDD, outperforming both conventional voting methods and several recently published approaches. These results indicate that class-specific F1-score-based weighting improves ensemble reliability, particularly under class imbalance, supporting its applicability to high-stakes classification tasks such as medical diagnosis.

## Introduction

Classification is a fundamental machine learning task with applications in image recognition, fraud detection, and medical diagnosis^1^. Medical diagnosis is a critical application of machine learning. Classification algorithms are used to identify diseases and conditions. These algorithms rely on various diagnostic data sources, such as medical imaging, patient records, and genetic information. Accurate classification is paramount, as misclassifications can lead to incorrect treatments or missed diagnoses, potentially endangering patients’ health^2^.

Ensemble learning, particularly voting-based strategies, is widely used in medical diagnosis because it helps improve the accuracy and robustness of predictions by combining the strengths of multiple classifiers^3,4^. However, challenges such as class imbalance, non-linear relationships between features, and noisy data can significantly impact the performance of traditional ensemble methods in medical domains. These challenges are particularly evident in datasets related to diseases like breast cancer, heart disease, and other life-threatening conditions, where the cost of misclassification is high^5^.

Voting-based ensemble methods, such as MV, WV, and SV, are widely used due to their simplicity^5^. However, they can struggle with non-linear datasets and multi-class classification, especially when the base models are not well-suited to capture complex relationships. These methods do not explicitly account for class-specific variations in classifier confidence, which can lead to suboptimal performance. While SV leverages probabilistic outputs, it does not dynamically adjust for class-specific performance, which may lead to poor results in imbalanced datasets, where majority class predictions dominate^6^.

Traditional voting methods assign static weights or rely on majority decisions, failing to account for classifiers excelling in certain classes while underperforming in others. MV treats all classifiers equally, WV relies on fixed weight assignments, and SV does not dynamically adapt based on class-specific confidence. These shortcomings reduce classification performance, especially in medical datasets where precision is critical^7^. An adaptive ensemble VS that adjusts dynamically based on class-specific reliability is necessary.

In this research, two real-world medical datasets are used to evaluate the effectiveness of the proposed dynamic voting strategies: the BCWD and the UHDD^8,9^. These datasets are commonly used benchmarks in machine learning and medical diagnostics, providing a rich source of data for developing and testing classification algorithms.

### Contributions of the study (contributions of the proposed work)

Existing research primarily focuses on fixed voting schemes, where classifier contributions remain unchanged across classes. An adaptive ensemble VS is presented with the following contributions:

- A class-scoring mechanism that assigns dynamic weights based on per-class F1-score.
- A refined SV strategy that dynamically adjusts classifier influence.
- Evaluation on synthetic datasets (Gaussian Mixture, Spiral, and Moon) and real-world medical datasets (BCWD & UHDD).
- Comparative analysis against traditional voting methods and recent ensemble approaches.

To guide the experimental investigation presented in this study, the following research questions are addressed:

- **RQ1**: Can class-specific F1-score-based voting strategies improve classification performance compared with conventional voting methods (Majority Voting, Weighted Voting, and Soft Voting) on non-linear and multi-class classification problems?
- **RQ2**: Among the proposed class-specific F1-score-based voting strategies (HCF1V, CCF1V, and ECF1V), which provides the most effective and consistent performance across datasets characterized by different levels of class imbalance, class overlap, and decision boundary complexity?

While adaptive weighting in ensemble learning has been explored in prior work, existing approaches predominantly assign weights at the classifier level rather than the class level. The present study extends this line of research by introducing per-class F1-score-based weight differentiation, where classifier k may carry high influence for class c₁ but low influence for class c₂, based on its validated performance. This distinction is particularly relevant in imbalanced multi-class settings where per-class discriminative reliability varies across classifiers.

### Paper organization

The remainder of this paper is structured as follows: Sect.  3 presents a detailed review of related works, discussing previous studies on ensemble learning in medical diagnosis and highlighting the limitations of existing voting strategies. Section  4 describes the proposed methodology, including the framework for class-scoring and dynamic weight assignment. Section  5 outlines the datasets used in the study, explaining the characteristics of both synthetic and real-world medical datasets. Section  6 provides experimental results and analysis, comparing the proposed VSs with traditional methods. Finally, Sect.  7 concludes the paper with key findings, limitations, and future research directions.

## Related works

Ensemble learning methods have been widely explored in classification tasks, particularly in the medical domain, where predictive accuracy is crucial. Traditional voting-based ensemble methods, such as MV, WV, and SV, have been commonly used; however, they exhibit limitations when applied to non-linear and imbalanced datasets as shown in Table 1.

Recent research has proposed various enhancements to ensemble methods to address these challenges, particularly in breast cancer and heart disease classification. For instance, an optimized stacking ensemble learning model has been developed for early breast cancer prediction, demonstrating improved performance over traditional classifiers^10^. Similarly, a voting ensemble machine learning approach combined with chi-square feature selection has been applied for early detection of cardiovascular diseases, showing significant improvements in predictive accuracy^11^. These advancements highlight the potential of refined ensemble techniques in enhancing classification performance in critical medical applications.

### Ensemble learning in breast cancer classification

Several studies have investigated the effectiveness of ensemble learning techniques in improving breast cancer detection accuracy.

A study by^12^ introduced an Adaptive Voting Ensemble approach, combining classifiers such as Extra Trees Classifier (ET), Light Gradient Boosting Machine (LGBM), Ridge Classifier (RC), and Linear Discriminant Analysis (LDA). The model dynamically adjusted classifier weights based on performance metrics, achieving an accuracy of 97.6% on the BCWD dataset. This approach demonstrated superior classification accuracy by effectively handling class imbalance through adaptive weight allocation.

Similarly^13^, proposed a MV Ensemble method, aggregating predictions from Support Vector Machine (SVM), K-Nearest Neighbors (KNN), Logistic Regression (LR), Decision Tree (DT), and Random Forest (RF) classifiers. The study achieved an accuracy of 98.1%, outperforming individual classifiers and highlighting the effectiveness of MV in breast cancer diagnosis.

Another comparative analysis by^14^ evaluated various machine learning models, including SVM, KNN, Neural Networks (NN), and Naïve Bayes (NB), to determine the most suitable model for breast cancer prediction. The study concluded that NNs (MLPClassifier) achieved the highest accuracy of 98.13%, followed by SVM and KNN. The results emphasized the importance of feature selection and hyperparameter tuning in improving classification performance.

Further^15^, explored the effectiveness of SV for breast cancer detection by integrating LR, SVM, and DTs. The study achieved an accuracy of 97.08%, confirming that probability-based aggregation methods enhance ensemble classification by incorporating model confidence levels.

Another relevant study by^16^ introduced a Majority-Based Voting Ensemble using Selective DTs trained on multiple datasets, including BCWD and UHDD. The study achieved 96.5% accuracy for breast cancer detection but exhibited lower performance (74.2%) on the heart disease dataset. The results suggested that while MV improves classification performance, it may struggle with complex or noisy datasets.

### Ensemble learning in heart disease classification

Heart disease prediction presents unique challenges due to non-linearity, class imbalance, and dataset variability. Recent research has explored various ensemble methods to improve classification accuracy in this domain.

A study by^17^ employed a Voting Classifier combining RF, Extreme Gradient Boosting (XGB), and SVM to enhance coronary artery disease (CAD) prediction. The model achieved an accuracy of 87%, demonstrating the effectiveness of ensemble learning in medical diagnosis.

Another study by^18^ introduced an Ensemble Classifier integrating KNN, SVM, and DT. The primary contribution was the use of a Multiple Feature Selection Algorithm (MFSA) to improve model performance. The ensemble model achieved 87.2% accuracy, highlighting the importance of feature selection in boosting classification accuracy.

Further advancements were presented in^19^, where a Stacked Ensemble method was implemented using NB, LR, and SVM, combined with an optimized Firefly Algorithm for feature selection. The study compared stacking and voting ensemble techniques, finding that stacking outperformed voting in heart disease classification, achieving 86.79% accuracy, while MV + SV reached 83.8% accuracy.

### Limitations of traditional voting methods

While ensemble methods improve classification performance, traditional MV, WV, and SV exhibit several limitations:

- Equal Weighting in MV: MV assumes that all classifiers contribute equally, which can lead to suboptimal predictions, especially when weak classifiers influence the outcome.
- Static Weights in WV: WV assigns static weights based on classifier performance, which may cause bias towards stronger classifiers, reducing diversity and increasing overfitting risk.
- Sensitivity to Probability Calibration in SV: SV depends on classifiers outputting well-calibrated probabilities, which is often difficult to achieve in real-world datasets.

The experimental results confirm that the proposed ECF1V and CCF1V outperform traditional ensemble methods, setting a new benchmark for medical diagnosis applications.

The proposed approach contributes to the growing body of research on dynamic voting strategies, offering an innovative approach to improving ensemble classification performance in challenging datasets.

**Table 1:** Comparison among recent related work on BCWD and UHDD datasets.

Ref	Voting Strategy	Algorithms Used	Dataset	Performance Metrics	Accuracy	Key Contribution
^12^	Adaptive Voting Ensemble	ET, LGBM, RC, LDA	BCWD	Accuracy, F1-score, Precision, Recall	97.6%	Improved breast cancer classification by dynamically adjusting classifier weights
^13^	MV Ensemble	SVM, RF, LR, DT, KNN	BCWD	Accuracy, Precision, Recall, F1-score, AUC	98.1%	Used MV to combine multiple classifiers and improve prediction
^14^	Not specified	SVM, KNN, NNs, NB	BCWD	Accuracy, Precision, Recall, F1-score, AUC-ROC	NN: 98.13%	Explored various ML models and hyperparameter tuning for breast cancer prediction
^15^	SV Classifier	LR, SVM, DT	BCWD	Accuracy, Precision, Recall, F1-score, AUC	97.08%	Investigated SV for improved breast cancer diagnosis accuracy
^16^	Majority-Based Voting Ensemble	Selective DT	BCWD, UHDD, and others	Accuracy, Precision, Recall	BCWD 96.5%, UHDD 74.2%	Developed a majority-based voting method using selective DTs for improved ensemble learning performance
^17^	Voting Classifier	RF, XGB, SVM	UHDD	Accuracy	87%	Enhanced heart disease prediction using multiple machine learning models with voting classifier ensemble
^18^	Ensemble Classifier	KNN, SVM, DT	UHDD	Accuracy	87.2%	Improved performance with feature selection (MFSA) in ensemble classifiers
^19^	Stacked Ensemble, MV + SV	NB, LR, SVM	UHDD	Accuracy	SD: 86.8%, MSV: 83.8%	Applied stacked ensemble with firefly feature selection for enhanced heart disease classification

## Methods - framework

Ensemble learning is a powerful machine learning approach that combines multiple classifiers to improve predictive performance and robustness. The SV strategy based on class scoring is designed to dynamically weight predictions from base classifiers by adjusting their influence based on confidence levels for each class [20]. Unlike traditional SV, which treats all classifiers equally, this method assigns weights dynamically, ensuring that classifiers with higher reliability for specific classes contribute more to the final prediction.

**Fig. 1:**
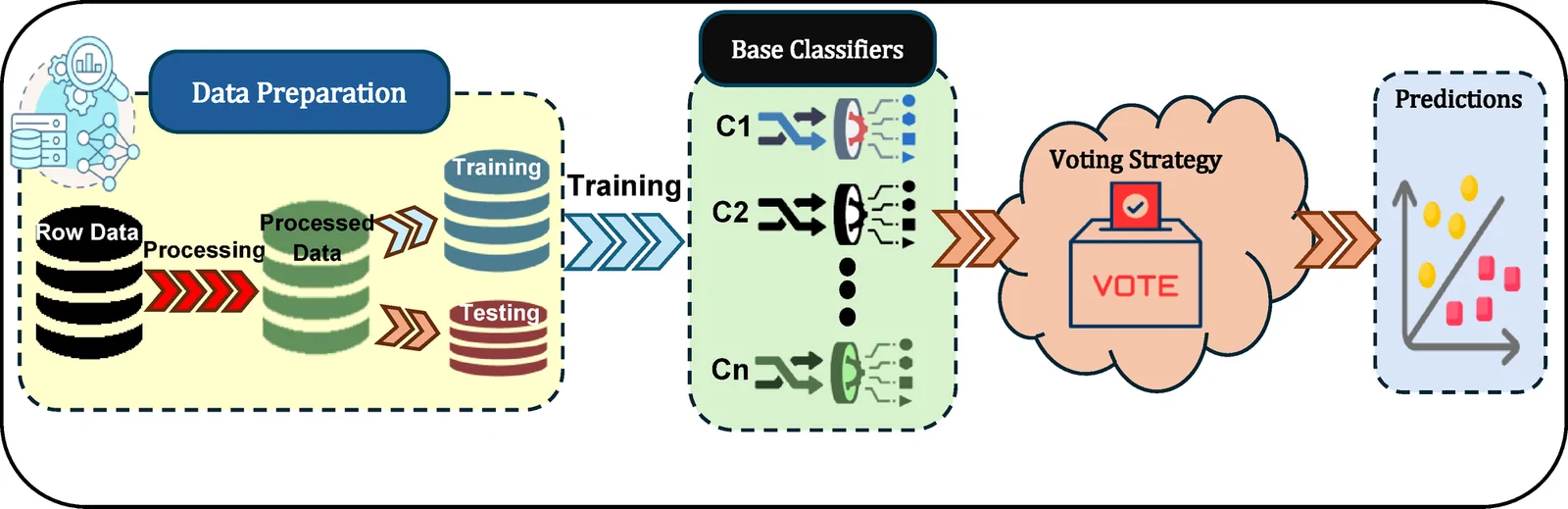
Ensemble classification voting framework.

Figure 1 illustrates the Ensemble Classification Voting Framework, which consists of four key stages: data preparation, classifier training, voting strategy, and prediction generation. The framework represents how multiple classifiers work together using a voting mechanism to improve classification performance.

The first stage, data preparation, involves processing raw data to obtain a refined dataset suitable for training and testing. This includes data cleaning, transformation, and feature selection. The processed data is then divided into training and testing sets, where the training data is used to develop classification models, and the testing data is reserved for evaluation.

The second stage involves Base Classifiers Training: A diverse set of classifiers (C1, C2, …, Cn) is trained on the processed dataset. Each classifier generates probabilistic predictions for all possible classes, providing a confidence score for each class in the dataset. Each classifier learns patterns independently and produces its own predictions. These classifiers may include various machine learning models such as DTs, SVM, LR, or ensemble methods like RF and gradient boosting.

Once the classifiers generate their predictions, the VS is applied to determine the final classification outcome. Common VSs include MV, where the most frequently predicted class is chosen, WV, where classifiers with higher accuracy have a greater influence, and SV, where probabilistic predictions are averaged for final decision-making. In this paper the voting phase includes:

### **C**lass scoring mechanism

To assess the reliability of each classifier for different classes, a class-specific confidence score is computed using performance metrics – in this study, the class F1-score – during the validation phase. These scores quantify how well each classifier performs for individual classes rather than considering overall accuracy. The scores are then normalized to create a dynamic weight matrix for each classifier, where a higher weight is assigned to classifiers that demonstrate stronger performance for a particular class.

A potential limitation of validation-based weight assignment is the risk of overfitting to the validation set, particularly when the validation data are limited in size or insufficiently representative of the underlying test distribution. Under such circumstances, the estimated per-class F1-scores may provide an overly optimistic assessment of a classifier’s true class-specific competence, potentially resulting in suboptimal weight assignments during inference. However, it should be noted that the proposed framework does not learn additional parameters through an optimization process; rather, the weights are directly derived from validation performance estimates, which inherently limits the degree of overfitting compared with parameter-learning ensemble methods. To mitigate this risk, a sufficiently large and stratified validation set should be employed whenever possible. In data-constrained scenarios, more robust estimation procedures, such as cross-validated or bootstrap-aggregated per-class F1-scores, may be adopted to improve the stability and reliability of the computed weights.

### Dynamic weight assignment

When making predictions, the probabilistic outputs from all classifiers are combined using the class-specific weight matrix. Instead of averaging the probabilities equally, each classifier’s contribution is scaled based on its performance for the respective class. The final prediction probability for a given class ccc is calculated as:

1 $$\:{P}_{final,\:c}={\sum\:}_{i=1}^{n}{\omega\:}_{i,\:c}\:.\:{P}_{i,\:c}$$

Where $\:{P}_{i,\:c}$ is the predicted probability for class * c* from classifier * i*, and $\:{\omega\:}_{i,\:c}$ is the class-specific weight.

More formally, the class-specific weights $\:{\omega\:}_{i,c}$can be organized into a weight matrix $\:{\Omega\:}\in\:{\mathbb{R}}^{K\times\:C}$, where * K*denotes the number of base classifiers and * C*denotes the number of classes. Each element $\:{\omega\:}_{k,c}$represents the validated per-class F1-score (or its normalized counterpart, depending on the weighting strategy) of classifier * k*for class * c*. This matrix-based representation fundamentally differentiates the proposed framework from conventional WV, which employs a single weight vector $\:\mathbf{w}\in\:{\mathbb{R}}^{K}$and therefore assigns the same classifier importance across all classes. In contrast, the proposed formulation enables class-dependent weighting, allowing a classifier to exert varying levels of influence for different classes according to its class-specific predictive performance. Consequently, the framework explicitly captures and exploits classifier specialization, providing the basis for its adaptive decision-making mechanism at the class level.

Finally, the prediction generation phase produces the final classification result. The final classification decision is determined by selecting the class with the highest aggregated probability score ($\:{P}_{final,\:c}$). This ensures that the decision is driven by classifiers that have proven to be more effective for each specific class, leading to improved predictive accuracy and robustness. By integrating multiple classifiers and leveraging a strategic voting mechanism, this framework enhances classification accuracy, reduces model bias, and improves overall predictive performance.

### Common voting strategies

Ensemble learning techniques leverage multiple classifiers to improve predictive accuracy and robustness. This section outlines common voting strategies, including MV, WV, and SV.

#### Majority voting (MV)

MV is a popular ensemble technique that helps reduce errors by combining the predictions of multiple classifiers. While it’s effective at lowering variance, it assumes that all classifiers are equally reliable and struggles with ties, especially in ensembles with an even number of classifiers. This simplicity is part of the reason why tools like scikit-learn’s VotingClassifier are so commonly used, as they make it easy to implement MV. However, this simplicity comes with some trade-offs. For example, MV treats all classifiers the same, which isn’t always realistic. In practice, different classifiers may perform better or worse depending on the dataset or class. So, when every classifier is given equal weight, the ensemble can be skewed by weaker or less accurate models, leading to suboptimal results [21].

The final prediction for a test instance $\:{x}_{i}$, determined by MV, is given by:

2 $$\:{\widehat{y}}_{i}=\mathrm{arg}{{max}}_{c\in\:C}{\sum\:}_{k=1}^{K}1\left[{\widehat{y}}_{k,\:i}=c\right]$$

where each classifier * k* contributes one vote.

Another issue with MV is that it doesn’t handle ties well. If there’s no clear majority vote—particularly with an even number of classifiers—the method has no built-in way to break the tie. This can lead to unpredictable results or require additional steps to make a decision, which adds complexity, especially in applications where the outcome needs to be reliable. Finally, MV treats all votes as equal, regardless of the confidence or probability behind each classifier’s prediction. This means it doesn’t take advantage of classifiers that are more confident or accurate in their predictions. In contrast, techniques like WV or SV consider the reliability and confidence of individual classifiers, offering a more nuanced and potentially more accurate final decision^22^.

#### Weighted voting (WV)

WV enhances MV by assigning weights to classifiers based on their performance metrics, such as accuracy. The final prediction is given by:

3 $$\:{\widehat{y}}_{i}=\mathrm{arg}{{max}}_{c\in\:C}{\sum\:}_{k=1}^{K}{\omega\:}_{k}1\left[{\widehat{y}}_{k,\:i}=c\right]$$

where $\:{\omega\:}_{k}$ represents classifier accuracy.

WV has proven effective in fields like medical imaging, cybersecurity, and regression tasks, where techniques like Relative Root Mean Square Error (RRMSE)-based weighting have improved accuracy^21^. It is also particularly efficient on nonlinear datasets, where traditional models may struggle, because assigning weights allows the ensemble to emphasize classifiers that perform better on specific parts of the data, thus handling complex patterns more effectively^23^.

However, WV has its own challenges. Its success relies heavily on accurate weight assignment—if the weights are wrong, it can skew the final prediction, especially if a weaker classifier gets too much weight. Additionally, WV tends to favor stronger classifiers, which can reduce diversity in the ensemble and even lead to overfitting if too much emphasis is placed on a few strong models. Also, determining the right weights can be complex and computationally expensive, requiring additional performance metrics or validation data. WV also assumes that classifiers perform consistently across different datasets, but in practice, their performance can fluctuate, reducing its effectiveness in dynamic environments^24^.

#### Soft voting (SV)

SV, or probability-based voting, averages class probabilities instead of discrete votes, leading to smoother and more confident predictions:

4 $$\:{\widehat{y}}_{i}=\mathrm{arg}{{max}}_{c\in\:C}\:\frac{1}{k}{\sum\:}_{k=1}^{K}{P}_{k}\left(c\:|\:{x}_{i}\right)$$

where $\:{P}_{k}\left(c\:|\:{x}_{i}\right)$ is the probability assigned by classifier * k* to class * c*.

SV has been applied successfully in fields like fraud detection, stress monitoring, and medical imaging, benefiting from improved classifier diversity and stability through techniques like bagging and random subspace selection^15^.

More recently, SV has been successfully integrated with transfer learning techniques in medical imaging applications. For example, ensemble-based frameworks have been developed for the detection of fundus lesion diseases from optical coherence tomography (OCT) images using soft-voting aggregation of deep learning models^25^. Such studies further demonstrate the continued effectiveness and practical relevance of voting-based ensemble methods in clinical decision-support systems.

However, SV has several limitations. One key challenge is that not all classifiers can output probabilities, such as decision trees or other non-probabilistic models. This may require additional techniques like Platt scaling or isotonic regression, adding complexity and potential unreliability. SV is also sensitive to poorly calibrated classifiers, where inaccurate probability estimates can distort the final decision. Additionally, SV can be biased toward the majority class in imbalanced datasets, as classifiers may assign higher probabilities to the more frequent class. Lastly, SV is computationally more expensive than MV, as it requires calculating probabilities for each class in each classifier, making it less efficient, especially for large datasets^20–22^.

It is worth noting that more advanced ensemble paradigms — including stacking, boosting, and dynamic ensemble selection (DES) — exist and have demonstrated strong performance across a range of classification tasks. However, the present study deliberately focuses on voting-based strategies, as the primary objective is to evaluate the effect of class-level performance weighting within a fixed ensemble structure. Introducing stacking or boosting would conflate the contribution of the voting mechanism with that of meta-learning or sequential reweighting, making it difficult to isolate the effect of the proposed weighting scheme. Comparison with DES methods, which share conceptual similarity with the class-level classifier selection underlying HCF1V, is identified as a meaningful direction for future work.

#### Proposed voting strategies

Three voting strategies based on class-specific F1-scores are presented in this section; the methodology, decision process, advantages, and limitations of each strategy are described.

The proposed voting strategies do not involve any form of instance injection, data augmentation, or modification of the training distribution. The handling of class imbalance is achieved entirely within the voting mechanism by assigning higher influence on classifiers that demonstrate stronger validated performance on minority classes, as reflected by their per-class F1-scores.

### Highest class F1-score-based voting (HCF1V)

**Fig. 2:**
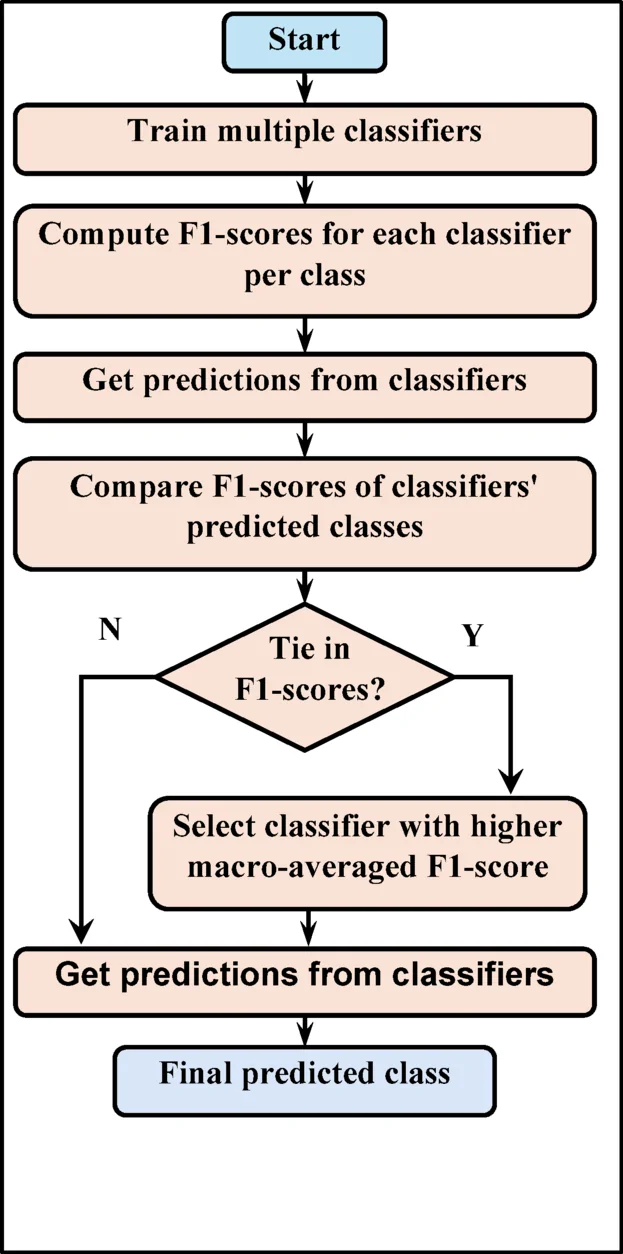
HCF1V Flowchart.

The HCF1V strategy is an ensemble classification method that selects the final prediction based on the classifier with the highest F1-score for each predicted class. Unlike MV or SV, which aggregate predictions from multiple classifiers, this approach dynamically chooses the most reliable classifier for each class, ensuring that the final decision is made by the model with the best class-wise performance. Figure 2 illustrates the decision process for HCF1V.

#### Methodology

For each test instance *x*_i_, classifiers *M*_*1*_, *M*_*2*_, *…*,* M*_*n*_ predict classes P(*x*_*i*_) = {p_1_, p_2_, …, p_n_} The final predicted class $\:{\widehat{y}}_{i}$ is determined as:

5 $$\:{\widehat{y}}_{i}=\mathrm{arg}{max}_{c\in\:P\left({x}_{i}\right)}\left({f}_{k,c}\right)$$

 

Where:

- $\:{f}_{k,c}$ is the F1-score of the classifier that predicted class c.

- The class with the highest F1-score among the predicted classes is selected.
- In rare cases, two classifiers may predict different classes with identical per-class F1-scores. The tie is resolved using the macro-averaged F1-score. The class predicted by the classifier with the higher macro-F1 score is selected.

The HCF1V strategy offers notable strengths for multi-class and imbalanced classification tasks. By selecting the classifier with the highest F1-score for each class, it ensures that the most competent model handles the final decision for that class. This improves class-specific accuracy and gives more balanced attention to minority classes, which are often overlooked in traditional ensemble methods. It also reduces the influence of weaker models, as only the best-performing classifier per class contributes to the final prediction.

However, HCF1V has several limitations. Relying on a single classifier per class removes the usual error-correction benefits of ensemble learning. If that classifier overfits or performs poorly due to noisy data, there’s no support from other models to compensate. The strategy may also struggle with overlapping classes, where insights from multiple classifiers could help resolve ambiguity but are ignored. Additionally, computing F1-scores for each classifier and class can be computationally expensive, especially with larger models or datasets. Performance may also vary between datasets, as the top classifier for one class in one dataset might not hold up in another. Overall, HCF1V is effective in specialized settings but may not be ideal for broader applications without careful consideration.

### Cumulative class F1-score-based voting (CCF1V)

**Fig. 3:**
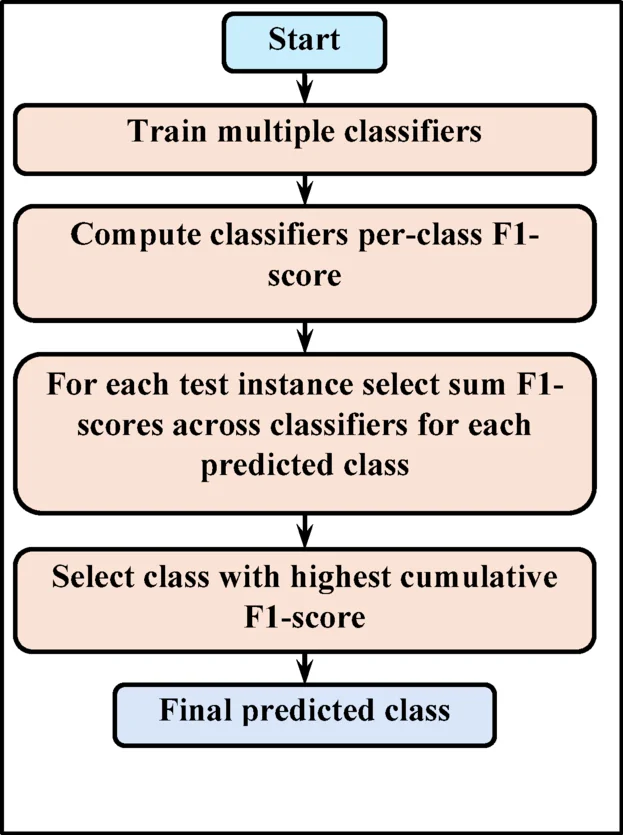
CCF1V Flowchart.

The CCF1V strategy enhances ensemble classification by incorporating F1-scores to weight predictions from multiple classifiers. Unlike traditional MV, where all classifiers contribute equally, this method prioritizes classifiers based on their class-specific F1-score performance. The goal is to aggregate votes in a way that emphasizes classifiers with better predictive accuracy for each class. Figure 3 illustrates the decision process for CCF1V.

#### Methodology

For each test instance *x*_i_, classifiers *M*_*1*_, *M*_*2*_, *…*,* M*_*n*_ predict classes P(*x*_*i*_) = {p_1_, p_2_, …, p_n_} The final predicted class $\:{\widehat{y}}_{i}$ is determined as:

6 $$\:{\widehat{y}}_{i}=\mathrm{arg}{{max}}_{c\in\:C}{\sum\:}_{k=1}^{K}{f}_{k,\:c}$$

Where:

- * C* is the set of all classes.
- * K* is the total number of classifiers.
- $\:{f}_{k,\:c}\:$is the F1-score of classifier * k* for class * c*.
- The prediction for $\:{x}_{i}$ is the class with the highest cumulative F1-score across all classifiers.

The Cumulative F1-Score-Based Voting (CCF1V) strategy offers several advantages in multi-class and imbalanced classification tasks. By incorporating the F1-score—which balances both precision and recall—it provides a more reliable metric for evaluating classifier performance, especially in situations where class distributions are skewed. Unlike majority voting (MV), which treats all classifiers equally, CCF1V dynamically weights predictions based on each classifier’s performance, giving more influence to those with a proven track record. This helps reduce the impact of weak classifiers, a common issue in traditional ensemble approaches.

However, CCF1V also presents some limitations. The method depends heavily on the quality and relevance of the F1-score, which may not always be the ideal metric—particularly in scenarios where either precision or recall is more critical. Additionally, the need to compute and maintain F1-scores per classifier and class adds computational complexity. There is also a risk of bias toward classifiers with high overall F1-scores, which can overshadow models that perform well on minority or harder-to-classify classes. Despite these challenges, CCF1V remains a robust and flexible ensemble strategy, particularly valuable when dealing with classifiers that have complementary strengths.

### Enhanced class F1-score-based voting (ECF1V)

**Fig. 4:**
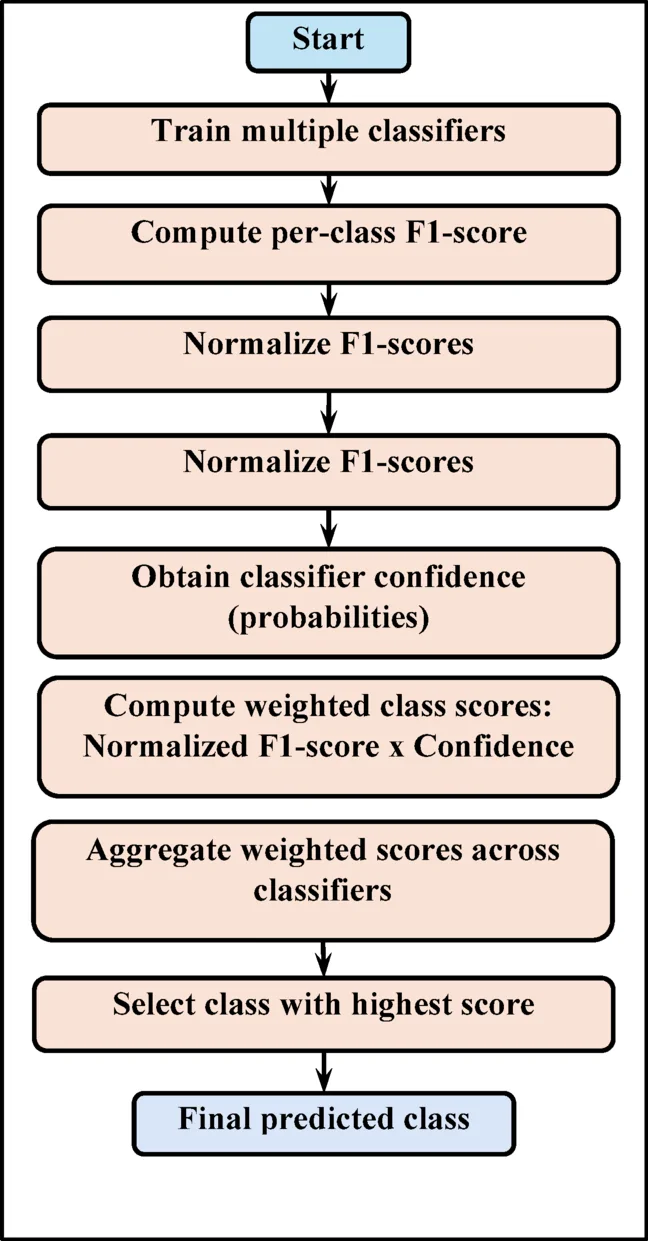
ECF1V Flowchart.

The ECF1V strategy refines traditional voting methods by incorporating normalized F1-scores and classifier confidence levels. This approach ensures that predictions are not only weighted by their performance on specific classes but also by how confident each classifier is in its predictions. By combining both factors, this method enhances reliability and reduces the influence of weak classifiers. Figure 4 illustrates the decision process for ECF1V.

#### Methodology

For each test instance $\:{x}_{i}$, the final predicted class $\:{\widehat{y}}_{i}$ is determined by summing the weighted contributions of all classifiers. The decision rule is mathematically defined as:

7 $$\:{\widehat{y}}_{i}=\mathrm{arg}{{max}}_{c\in\:C}{\sum\:}_{k=1}^{K}{f}_{k,\:c}\:\frac{{f}_{k,c}}{{\sum\:}_{j\in\:c}{f}_{k,j}}\:.\:{P}_{k}\left(c\:|\:{x}_{i}\right)$$

Where:

- * C* is the set of all classes.
- * K* is the total number of classifiers.
- $\:{f}_{k,\:c}\:$is the F1-score of classifier * k* for class * c*.
- $\:\frac{{f}_{k,c}}{{\sum\:}_{j\in\:c}{f}_{k,j}}\:$is the normalized F1-score for class * c* from classifier * k*.
- $\:{P}_{k}\left(c\:|\:{x}_{i}\right)$ is the confidence (predicted probability) of classifier * k* for class * c*.

The ECF1V strategy offers a powerful and nuanced approach to ensemble learning by integrating both historical performance and real-time prediction certainty. One of its core strengths lies in its ability to balance classifier performance and confidence, ensuring that final predictions are made not only by the most historically accurate models (as indicated by F1-scores) but also those most confident in their current predictions. This combination significantly enhances decision reliability, especially in complex or uncertain classification tasks.

Unlike simpler ensemble methods, ECF1V normalizes per-class F1-scores to limit the influence of classifiers that perform poorly across classes. Additionally, this strategy is highly effective in addressing class imbalance, a common challenge in real-world datasets, as it dynamically adjusts weights to ensure minority classes are adequately represented. The use of confidence scores also promotes robust and stable decision-making, especially when classifiers disagree, helping the ensemble resolve ambiguous cases more effectively. ECF1V is particularly well-suited for use in high-stakes domains, such as medical diagnosis, fraud detection, and financial risk assessment, where both predictive accuracy and decision certainty are critical.

Furthermore, its ability to leverage the diverse strengths of multiple classifiers—allowing each to contribute based on both prior performance and present certainty—makes it highly adaptable to a range of ensemble configurations and real-world applications. While the method does introduce additional computational complexity, the gains in reliability, interpretability, and class-level fairness make ECF1V a compelling strategy for demanding classification tasks.

The ECF1V strategy relies on classifier probability outputs, and its performance may be sensitive to probability miscalibration. Classifiers such as Decision Trees and Naïve Bayes are known to produce poorly calibrated probability estimates. While the normalization of F1-scores in ECF1V provides a degree of robustness by down-weighting unreliable classifiers, applying explicit calibration techniques such as Platt scaling or isotonic regression prior to voting represents a promising direction for future work.

In a related direction, correlation-based attribute weighting has been proposed to enhance the robustness and predictive reliability of Naïve Bayes classifiers by adjusting feature contributions according to inter-attribute correlations^26^. While such approaches operate at the feature level, the proposed framework performs adaptation at the class level through performance-driven ensemble weighting. Exploring the integration of feature-level calibration techniques with the proposed class-specific F1-score weighting scheme may represent a promising avenue for future research, potentially improving both classifier reliability and ensemble-level decision quality.

**Fig. 5:**
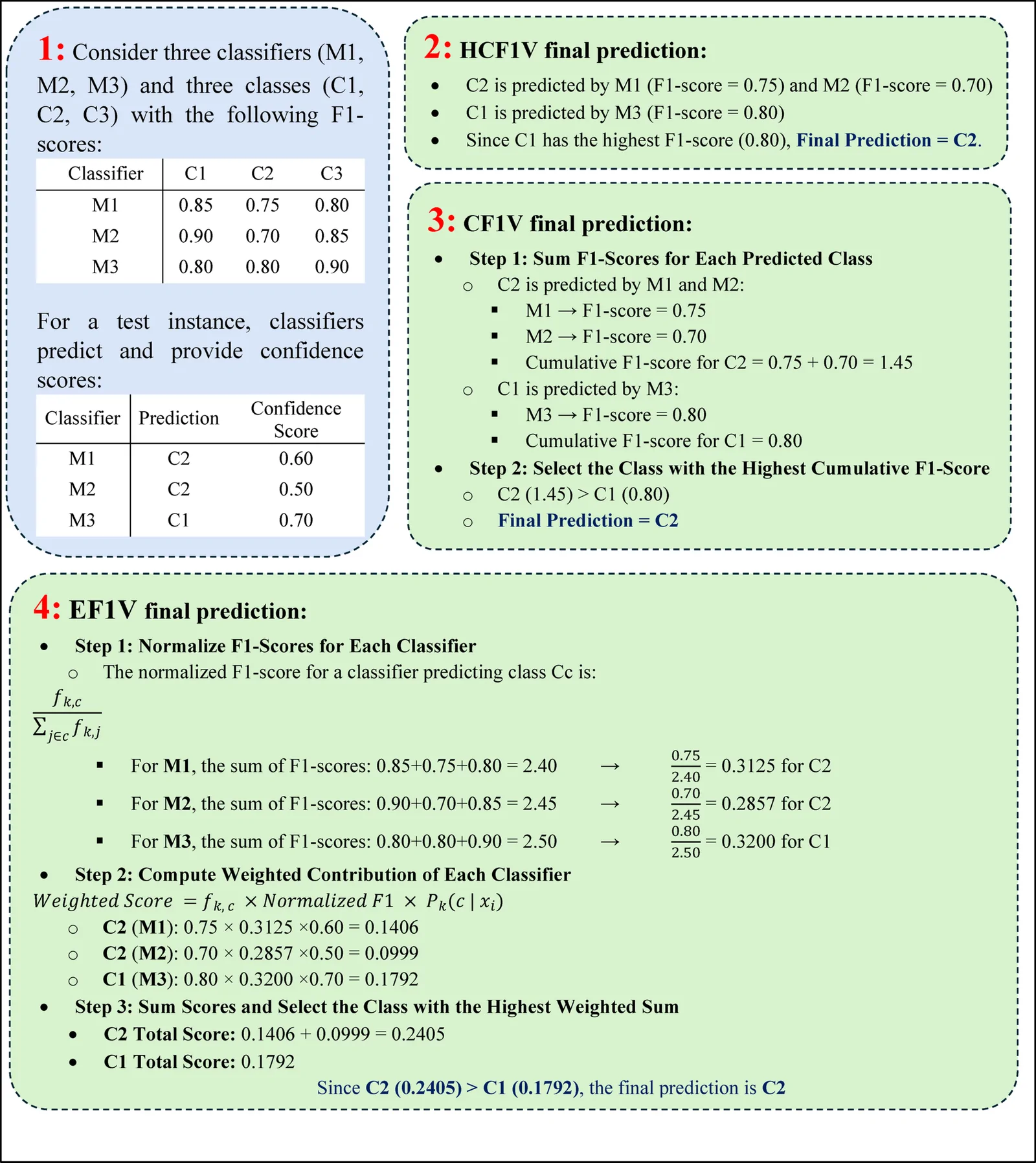
Example: to demonstrate how final prediction is estimated for each proposed voting strategy.

**Fig. 6:**
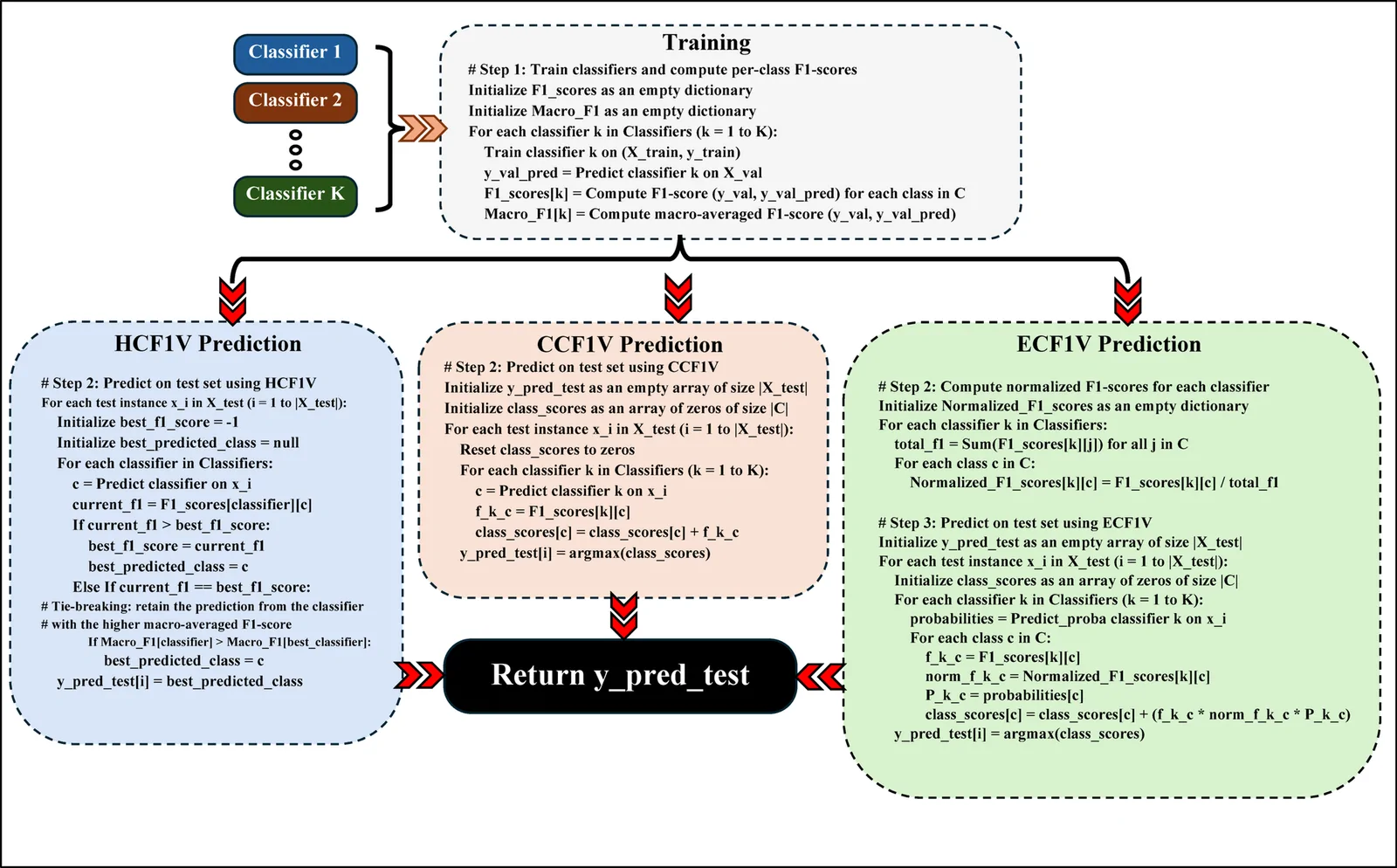
Proposed voting strategies pseudocodes.

Figure 5 demonstrates an example of how final prediction is estimated for each proposed voting strategy, while Fig. 6 illustrates the pseudocodes of the proposed voting strategies. Table 2 represents a comparison among proposed voting strategies, while Table 3 shows a comparison between common and proposed voting strategies.

The computational complexities of the three fusion strategies differ substantially. **HCF1V** has a time complexity of **O(KN)** because, for each test instance, it evaluates only the * K*classifier predictions without iterating over all $\:\mid\:C\mid\:$classes. In contrast, **CCF1V** has a complexity of **O(KN|C|)**, as it aggregates class-specific F1-scores across all classifiers and classes for each instance. Similarly, **ECF1V** also exhibits a complexity of **O(KN|C|)**; however, it incurs a larger constant factor due to the additional normalization term $\:{\sum\:}_{j\in\:C}{f}_{k,j}$computed for each classifier and the incorporation of the confidence probability $\:{P}_{k}(c\mid\:{x}_{i})$into the weighting process. Here, * K*denotes the number of classifiers, * N*the number of test instances, and $\:\mid\:C\mid\:$the number of classes.

For reference, conventional Soft Voting exhibits the same inference-time complexity, **O(KN|C|)**, as both CCF1V and ECF1V. The additional computational cost introduced by ECF1V is limited to a small constant-factor overhead associated with F1-score normalization and confidence-based weighting. These operations are computationally inexpensive and, in practice, did not produce any statistically meaningful runtime differences across the datasets evaluated in this study.

Regarding classification performance, both **CCF1V** and **ECF1V** demonstrate improved sensitivity to minority classes. This improvement arises because class-specific F1-scores capture the precision–recall balance achieved by each classifier for a given class. Consequently, classifiers that achieve stronger performance on minority classes—particularly higher recall—receive proportionally greater influence in the voting process, thereby increasing the likelihood that correct minority-class predictions are reinforced in the final ensemble decision.

Finally, the proposed Class F1-Score Voting Method offers significant advantages over traditional ensemble voting approaches, especially for imbalanced datasets and multi-class classification problems. Unlike conventional methods that use fixed weights, this approach dynamically adjusts the influence of each classifier based on its class-specific F1-score. By prioritizing classifiers that excel in predicting specific classes, it reduces misclassification rates and improves overall accuracy. This adaptability is particularly useful when different classifiers perform well on different categories, ensuring balanced and precise predictions.

Additionally, the method is highly flexible and can be easily integrated into various ensemble frameworks and classifier architectures, such as RF, Gradient Boosting, and NNs. This versatility makes it a robust solution for complex tasks, especially those with skewed class distributions. By leveraging class-specific performance metrics, the Class F1-Score Voting Method optimizes classifier contributions, delivering more reliable and accurate results compared to traditional hard-voting and soft-voting mechanisms.

**Table 2:** Comparison among the three proposed VSs across key design criteria.

Criteria	HCF1V	CCF1V	ECF1V
**Decision Mechanism**	Selects prediction from classifier with highest F1-score for each class	Sums F1-scores from all classifiers and selects the class with the highest total	Combines normalized F1-scores with classifier confidence
**F1-Score Usage**	Uses only the highest F1-score per class	Uses cumulative F1-scores from all classifiers	Uses normalized F1-scores
**Incorporates Classifier Confidence**	No	No	Yes
**Handles Class Imbalance**	Yes	Yes	Yes
**Reduces Weak Classifiers’ Influence**	Yes	Moderate	Yes
**Risk of Bias**	High Relies on a single classifier per class, prone to errors if that classifier is weak.	Moderate Summing F1-scores helps balance decisions, but dominant classifiers may overpower minority classes.	Low Incorporates confidence scores, reducing the impact of biased classifiers.
**Computational Complexity**	Low	Medium	High
**Best Use Case**	Multi-class classification where some classifiers excel in specific classes	Imbalanced datasets where multiple classifiers contribute to class distinction	Critical applications (e.g., medical diagnosis, fraud detection) requiring both accuracy and confidence

**Table 3:** Comparison between conventional voting strategies (MV, WV, SV) and the three proposed F1-score-based strategies.

Voting Strategy	Description	Strengths	Weaknesses	Best Use Cases
**MV**	Each classifier votes for a class, and the majority class is chosen.	Simple, interpretable, reduces variance.	Assumes equal classifier reliability, struggles with ties.	General ensemble classification, reducing variance.
**WV**	Classifiers are weighted based on their accuracy or performance metrics.	Accounts for model performance, reduces influence of weak classifiers.	Relies on accurate weight assignment, biased towards strong classifiers.	Regression and classification with model performance variation.
**SV**	Averages predicted probabilities across classifiers and selects the highest.	Smooths predictions, provides confidence estimates, handles uncertainty.	Requires probability outputs, sensitive to poorly calibrated models.	Imbalanced datasets, cases requiring probability confidence.
**HCF1V**	Selects the prediction from the classifier with the highest F1-score for each class.	Improves class-specific accuracy, ideal for imbalanced data.	Over-relies on a single classifier per class, less robust to noise.	Multi-class classification with imbalanced datasets.
**CCF1V**	Sums F1-scores of all classifiers for each class and selects the highest.	Balances precision and recall, mitigates weak classifiers’ influence.	Over-depends on F1-score, may underrepresent minority class classifiers.	Ensembles where classifiers have varied strengths per class.
**ECF1V**	Normalizes F1-scores and incorporates classifier confidence in decision-making.	Combines historical performance and confidence, adaptable to imbalanced data.	High computational cost, requires normalization and confidence estimation.	High-confidence applications like medical diagnostics and fraud detection.

The proposed framework does not employ SMOTE, duplication, interpolation, or any other oversampling or undersampling technique^27^. The training and validation sets are used in their original class distributions. The improved handling of minority classes is an emergent property of the class-level F1-score weighting applied at the prediction stage.

## Datasets

**Fig. 7:**
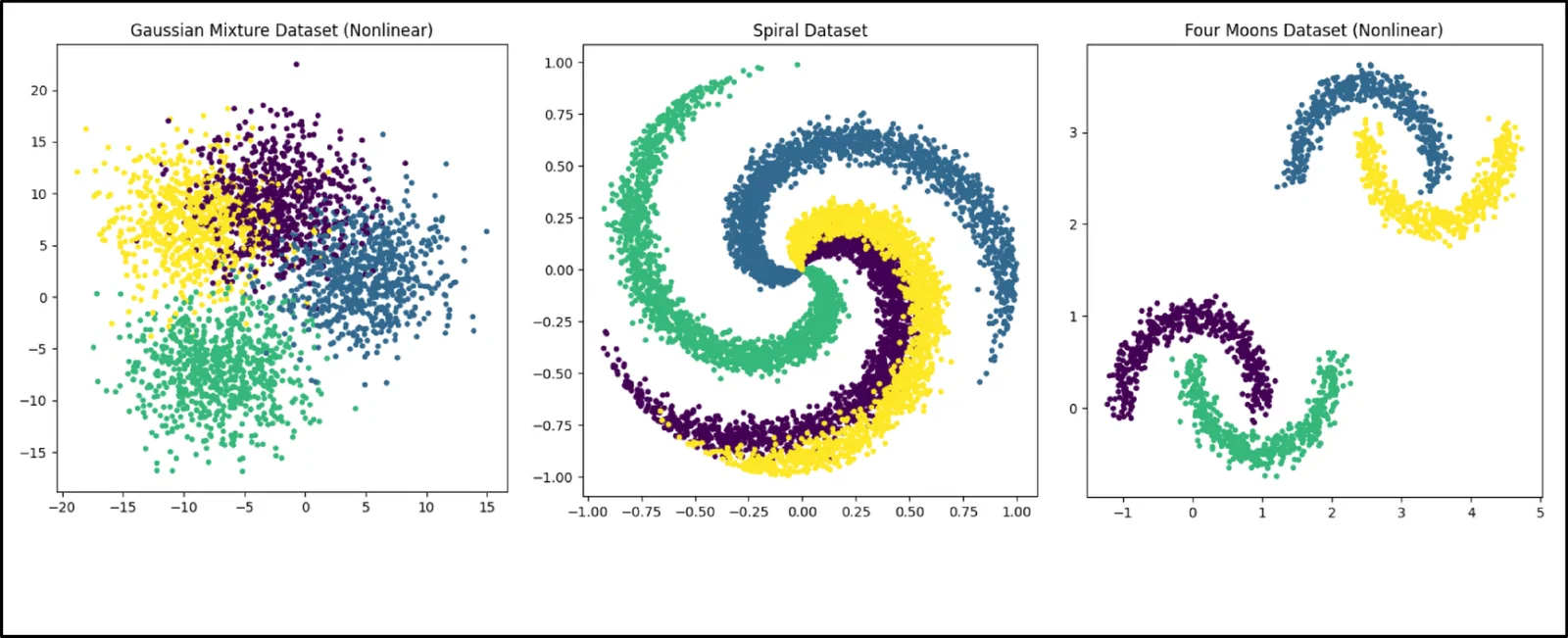
Generated datasets visualization.

Five datasets are used to evaluate the proposed voting strategies: three randomly generated datasets (**Gaussian mixture Dataset**,** Spiral Dataset**,** Moon Dataset**) used to compare between common and proposed voting strategies, on the other hand, two well-known datasets (**BCWD**, **UHDD**) used to compare the proposed VSs with recent research works. Figure 7 shows the visualization of the generated datasets.

### Gaussian mixture dataset (non-linear)

The Gaussian mixture Dataset generated consists of 3,000 data points, divided into four distinct clusters, with each cluster representing a different class. Using the make_blobs function from scikit-learn, the points are spread across a two-dimensional space, with a standard deviation of 3.5 around each cluster center, creating a noticeable dispersion of points within each group. The clusters are well-separated, though with some overlap due to the chosen standard deviation, allowing for a more challenging classification or clustering task^28^.

The dataset is created with a random state of 42 (as commonly used in machine learning for reproducibility purposes), ensuring reproducibility of the points’ distribution. Each data point is assigned one of four class labels, ranging from 0 to 3, corresponding to the cluster to which it belongs. This synthetic dataset is ideal for evaluating and comparing various machine learning algorithms, particularly those focused on classification and clustering, providing a controlled but complex scenario for testing model performance in classifying or clustering well-defined groups^28^.

### Spiral dataset

The spiral dataset generated consists of 8,000 data points, divided into four distinct classes, each represented by a spiral pattern. The dataset was created using a custom function, where each class corresponds to a separate spiral, and the points are distributed in a two-dimensional space. For each class, the radial coordinate is linearly spaced between 0 and 1, while the angular coordinate spans a range determined by the class number, with added Gaussian noise to introduce variability and complexity. This results in spirals that wind around the origin, with the data points for each class forming a distinct, curved shape. The dataset is designed to be non-linearly separable, posing a challenge for traditional classification algorithms that rely on linear decision boundaries.

The class labels range from 0 to 3, with each class containing 2,000 points. This spiral dataset is ideal for evaluating the performance of machine learning algorithms, particularly those capable of handling complex, non-linear decision boundaries such as kernel methods or deep learning models. The structure of the dataset makes it a useful tool for testing classification techniques in scenarios where traditional methods struggle with separation due to the intricate pattern of the data.

### Moon dataset

The moon dataset generated consists of 2,000 data points, divided into four distinct classes, each represented by a moon-shaped cluster. The dataset is created by combining two sets of moon-shaped clusters, each containing 1,000 points, and shifting one of the sets to introduce spatial separation between the clusters. The points in the first set of moons are generated using the make_moons function, while the second set is similarly created but shifted by 2.5 units in both the x and y coordinates. The class labels are then modified to create four classes: the first set of moons is assigned labels 0 and 2, while the second set is assigned labels 1 and 3^29^.

The resulting dataset is non-linearly separable, with interleaving moon-shaped structures that challenge traditional classification methods. The addition of noise (with a value of 0.1) further complicates the task by introducing small variations in the data points. This dataset is well-suited for evaluating the performance of machine learning algorithms on complex, non-convex decision boundaries, particularly in scenarios involving noisy and imbalanced class distributions. The four moons dataset provides a useful benchmark for testing the ability of classifiers to distinguish between intricately structured, non-linearly separable clusters^29^.

### Breast cancer wisconsin (diagnostic) dataset (BCWD)

BCWD is a well-known dataset from the UCI Machine Learning Repository, originally donated in 1995^30^. It contains 569 instances, with 30 features for each sample, and the goal is to classify whether a breast cancer tumor is malignant (M) or benign (B). The features in the dataset are derived from a digitized image of a fine needle aspirate (FNA) of a breast mass. These features describe the characteristics of the cell nuclei present in the image, including metrics like radius, texture, smoothness, compactness, concavity, and others.

The dataset does not contain missing values and is used primarily for binary classification tasks. The target variable is the diagnosis, with two possible classes: *M* for malignant and *B* for benign. The features are real-valued and help in building predictive models for early diagnosis of breast cancer. It is often used as a benchmark in evaluating machine learning algorithms for classification.

**Fig. 8:**
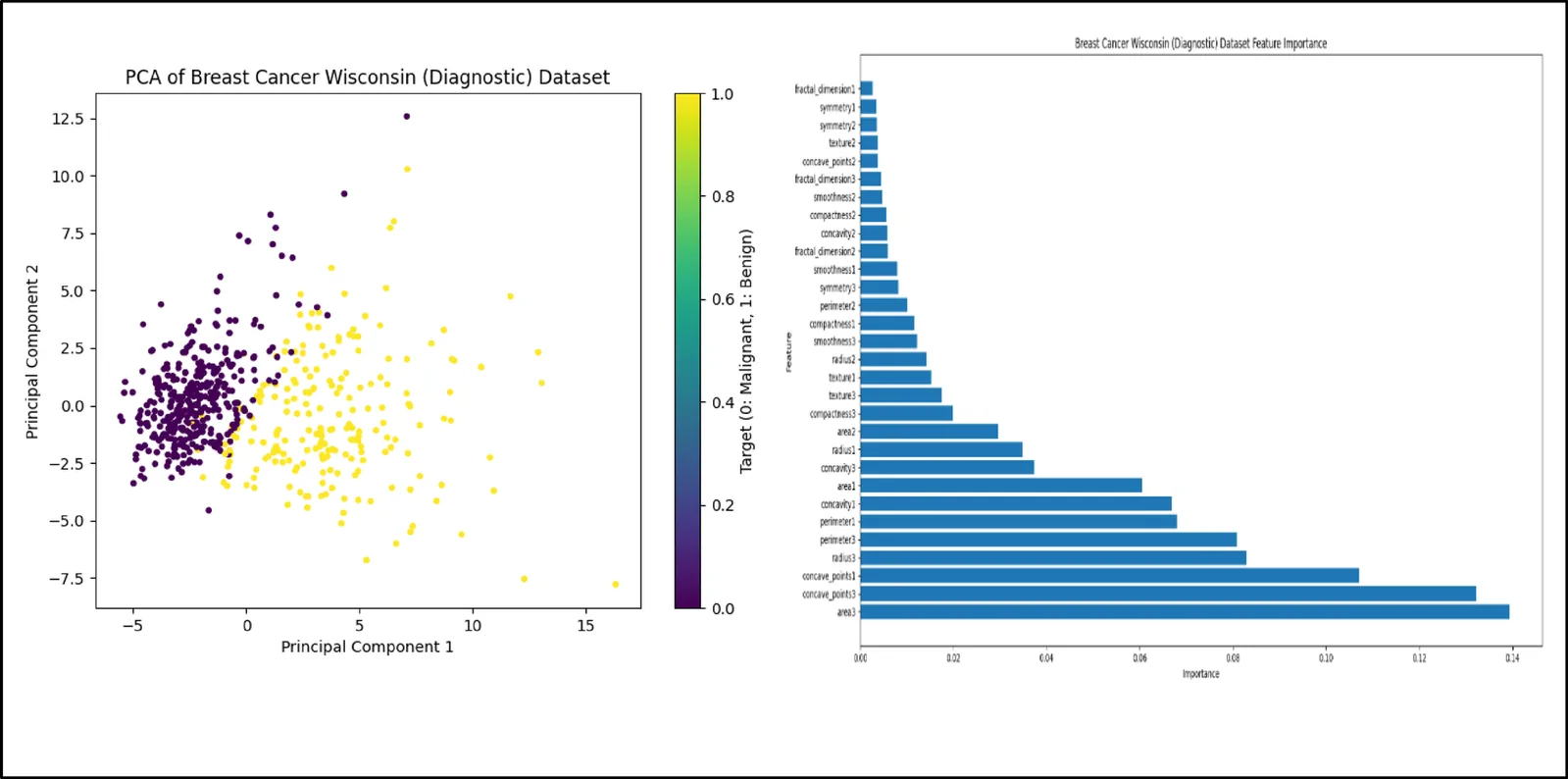
BCWD visualization and feature importance.

Figure 8 presents the visualization and feature importance of various attributes in the BCWD Dataset, highlighting their relative contributions to the predictive model. The horizontal bar chart ranks features based on their importance, with area3, concave_points3, and concave_points1 emerging as the most influential factors. These features have the highest importance scores, suggesting that characteristics related to tumor area, concavity, and perimeter play a crucial role in distinguishing between malignant and benign tumors. Conversely, features such as fractal_dimension1, symmetry1, and symmetry2 exhibit the lowest importance, indicating their relatively minor contribution to the model’s predictive capability. This analysis aligns with existing medical research, which emphasizes the significance of tumor shape and size in breast cancer diagnosis. Understanding feature importance helps in optimizing predictive models by focusing on the most relevant variables, potentially improving classification accuracy while reducing computational complexity.

**Fig. 9:**
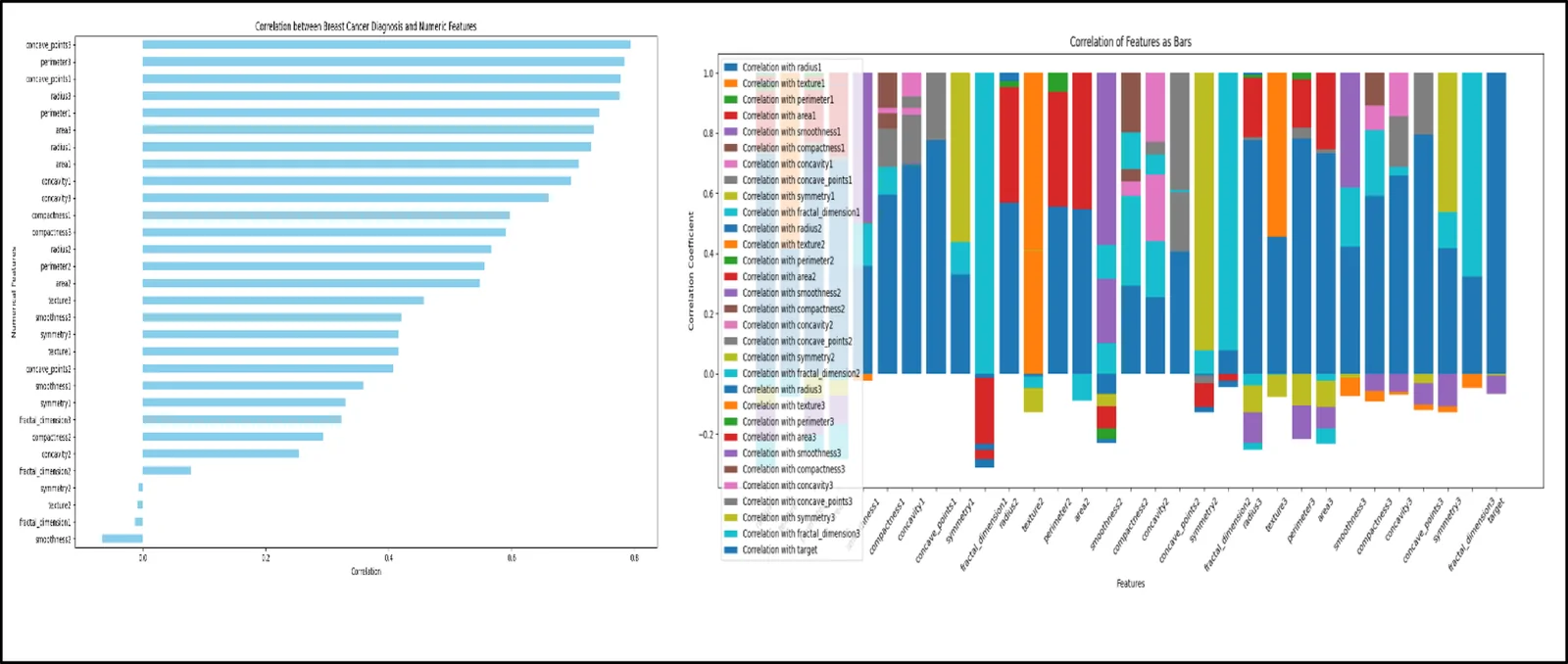
BCWD Features Correlation.

Figure 9 provides insights into the correlations between various features in the Breast Cancer Wisconsin (Diagnostic) dataset and the target variable (diagnosis). Features such as concave points and radius/perimeter are highly correlated with the target, suggesting they are strong indicators of malignancy. In contrast, features like compactness, smoothness, and fractal dimension show weaker correlations, indicating they may contribute less to distinguishing between benign and malignant tumors. Additionally, features related to tumor size, such as radius_1 and perimeter_1, exhibit strong correlations with each other, highlighting their redundancy. These findings underscore the importance of shape and size-related features, particularly concave points, for accurate breast cancer diagnosis, while features like fractal dimension may have less predictive power.

### UCI heart disease dataset (UHDD)

HDD from the UCI Machine Learning Repository contains data on 303 patients, with 13 features used for predicting the presence of heart disease. These features include demographic information such as age and sex, as well as medical attributes like cholesterol levels, blood pressure, and exercise-induced angina. The goal is to predict whether a patient has heart disease, with the target variable being binary, indicating the presence (values 1–4) or absence (value 0) of the disease^31^.

The dataset includes categorical, integer, and real-valued features. Missing values are present, and the dataset has been widely used for classification tasks, offering a benchmark for evaluating various machine learning algorithms in the health and medicine domain. The Cleveland database is the most commonly used subset for research, although other versions (Hungary, Switzerland, and VA Long Beach) are also available.

**Fig. 10:**
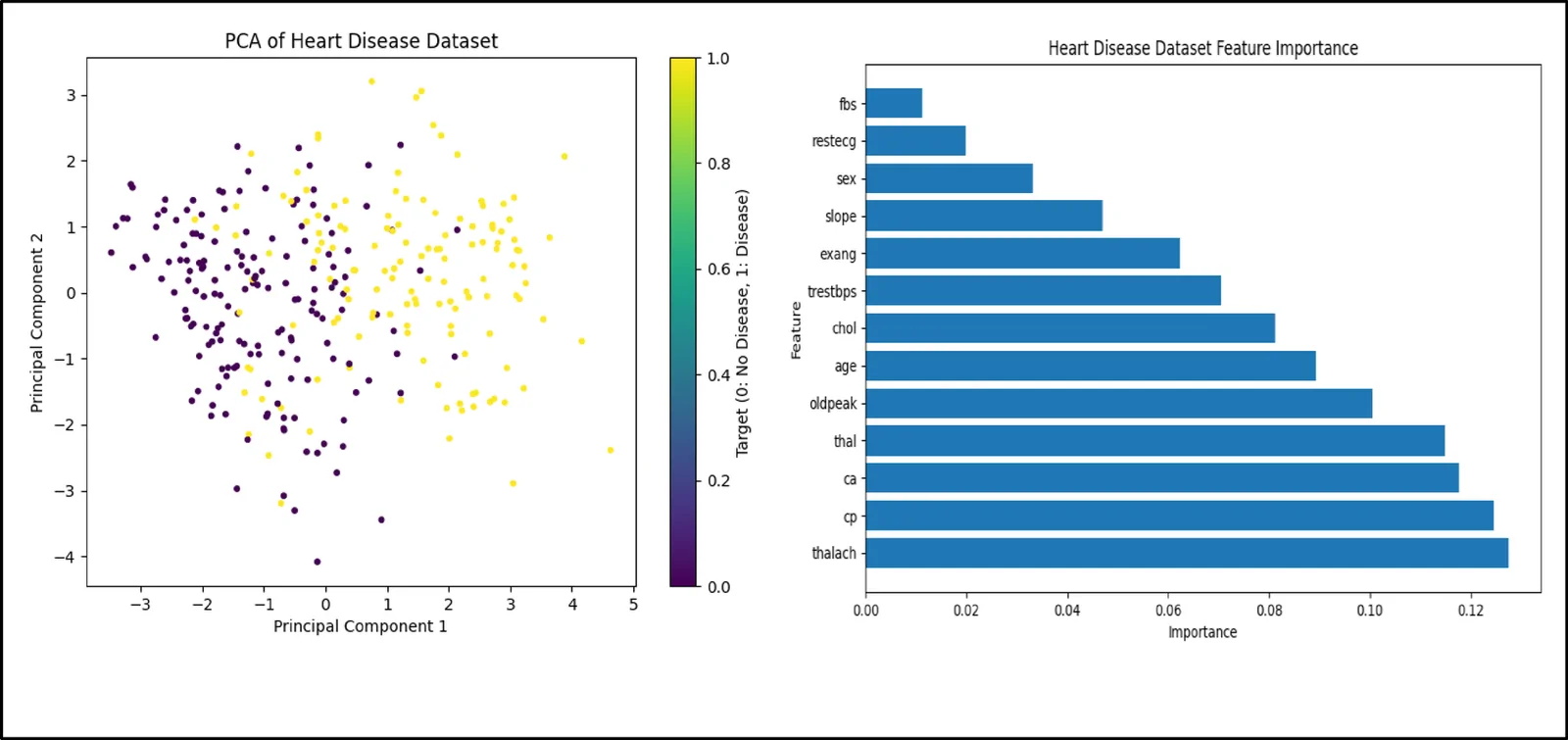
UHDD Visualization and Feature Importance.

Figure 10 illustrates the visualization and feature importance of various attributes in the Heart Disease Dataset, highlighting their relative contributions to the predictive model. The horizontal bar chart ranks features based on their importance, with thalach (maximum heart rate achieved), chest pain type (cp.), and ca. (number of major vessels colored by fluoroscopy) emerging as the most influential predictors of heart disease. These features exhibit the highest importance scores, suggesting a strong correlation between cardiovascular stress response, arterial blockage, and the likelihood of heart disease.

Additionally, features such as thal (thalassemia), oldpeak (ST depression induced by exercise), and age also contribute significantly to the model’s predictive performance. Conversely, attributes like fasting blood sugar (fbs), resting electrocardiographic results (restecg), and sex demonstrate lower importance, indicating a comparatively lesser impact on heart disease prediction. These insights align with clinical findings that factors such as exercise-induced stress, chest pain, and arterial abnormalities are key indicators of cardiovascular health. Understanding feature importance aids in refining predictive models, improving diagnostic accuracy, and guiding clinical decision-making by prioritizing the most relevant variables.

**Fig. 11:**
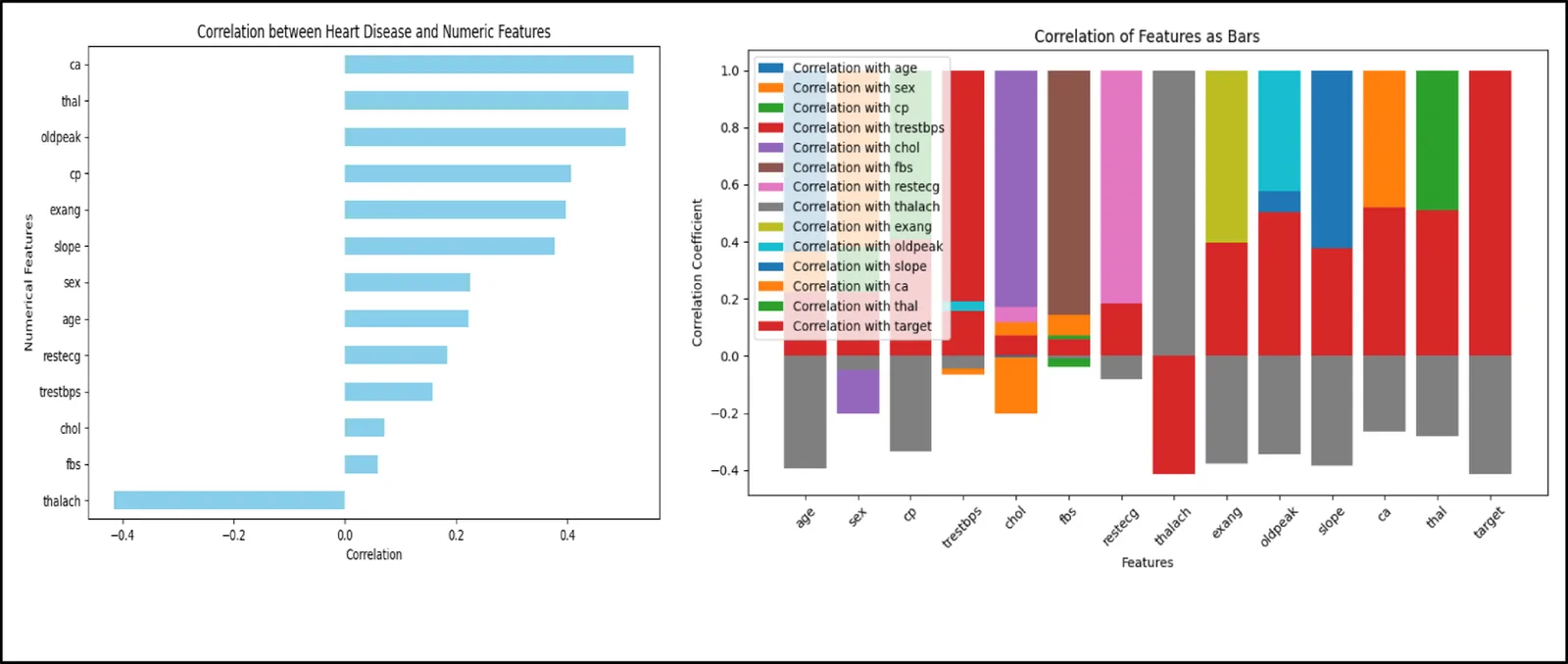
UHDD Features Correlation.

Figure 11 provides insights into the correlations between various features in the UHDD and heart disease. The left chart highlights that features such as ca. (Number of Major Vessels), thal (Thalassemia), oldpeak (ST Depression induced by exercise), and cp. (Chest Pain Type) have strong positive correlations with the presence of heart disease, suggesting their importance in prediction. On the other hand, thalach (Maximum Heart Rate Achieved) exhibits a negative correlation, indicating that higher heart rates are linked to lower risk. The right chart further illustrates feature interactions, showing that age, chol (Serum Cholesterol), and restecg (Resting Electrocardiographic Results) are strongly correlated, while features like fbs (Fasting Blood Sugar) and thal exhibit weaker relationships with other features. Overall, these findings underscore the significance of certain features, such as ca., thal, and oldpeak, in heart disease prediction, while others, like thalach, serve as negative predictors.

## Results and discussion

This section presents the outcomes of the experiments conducted on the five datasets discussed in the previous section. The performance of various voting strategies, including both common ensemble methods and the proposed F1-score-based approaches, was evaluated across these datasets to assess their classification effectiveness. The results highlight the impact of different voting mechanisms on predictive accuracy, robustness, and handling of class imbalances. Comparative analyses of Receiver Operating Characteristic (ROC) curves, Area Under the Curve (AUC) values, and accuracy metrics provide insights into the relative advantages of each method.

### Experimental setup and reproducibility

- **Random seed**: All experiments used a fixed random state of 42 for reproducibility.
- **Data preprocessing**: Features were normalized using min-max scaling for KNN and SVM-based models; no normalization was applied for tree-based classifiers. Missing values in UHDD were handled by median imputation.
- **Train-test split**: An 80/20 stratified split was used for all datasets, with a separate 20% validation split drawn from the training set for F1-score computation.
- **Hyperparameters**: Default scikit-learn parameters were used for all base classifiers. No dataset-specific hyperparameter tuning was applied, to ensure a fair comparison of voting strategies rather than individual classifiers.
- **Hardware/software**: Experiments were conducted on a machine with an Intel Core i7 processor, 16 GB RAM, running Python 3.10 with scikit-learn 1.3.

The following subsections detail the experimental findings for each dataset, emphasizing the strengths and limitations of different VSs in diverse classification tasks. For the evaluation on synthetic datasets, we employed SVM, KNN, NB, and DT as the base classifiers for the proposed voting strategies. SVM, KNN, NB, and DT offer diverse strengths: SVM excels at handling non-linear problems, KNN captures local patterns, NB works well with probabilistic and imbalanced data, and DT is interpretable and handles both numerical and categorical data.

### Evaluation on gaussian mixture dataset

**Fig. 12:**
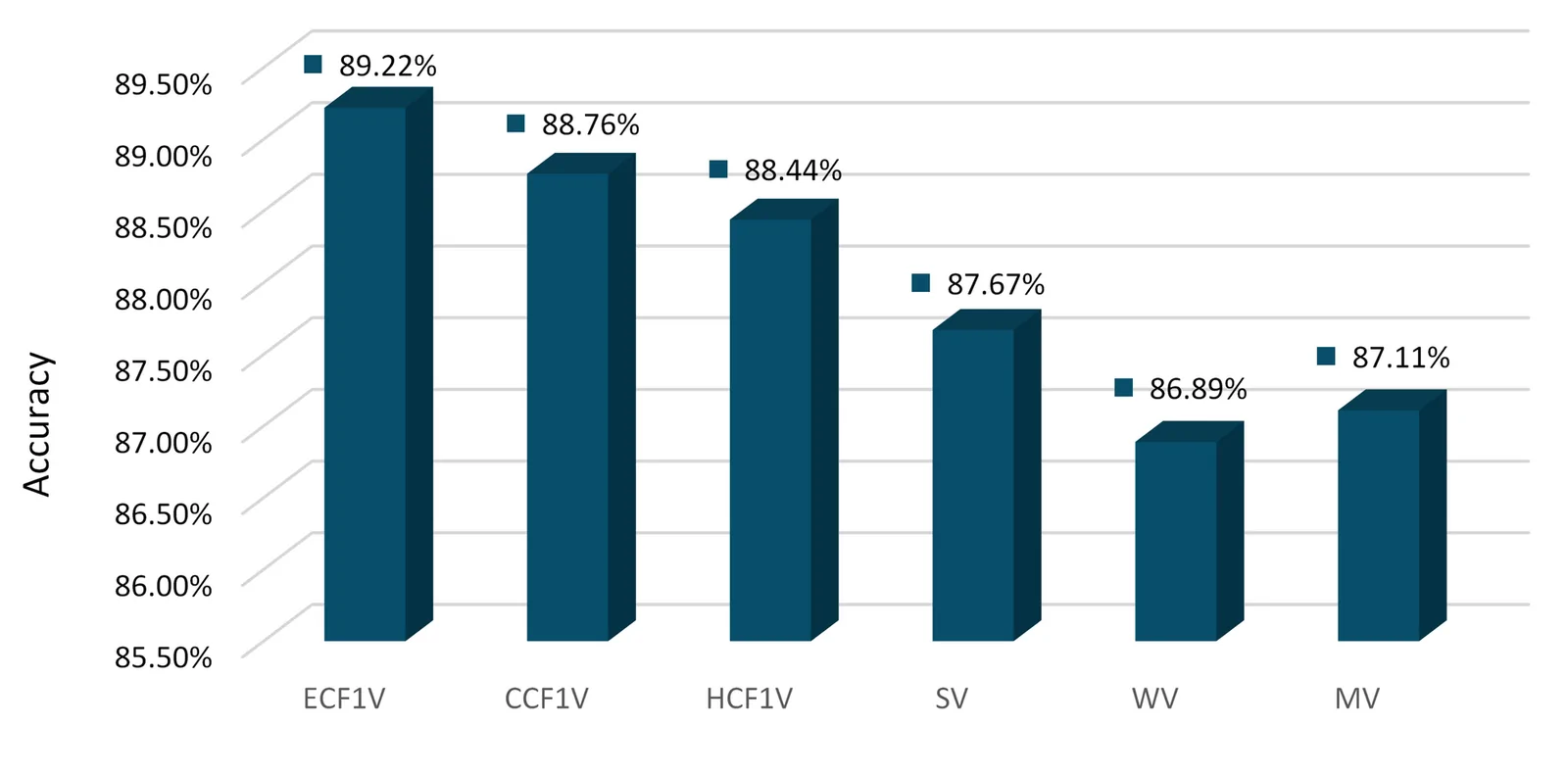
Classification accuracy of six voting strategies evaluated on the Gaussian Mixture Dataset.

Figure 12 illustrates the accuracy of different VSs applied to the Gaussian Mixture Dataset, comparing their effectiveness in ensemble classification. The bar chart presents six voting strategies: ECF1V, CCF1V, HCF1V, SV, WV, and MV. Among these, ECF1V achieves the highest accuracy at 89.22%, followed closely by CCF1V (88.76%) and HCF1V (88.44%), indicating that the proposed F1-score-based VSs (F1S-VS) enhance classification performance.

Traditional methods, such as MV (87.11%) and WV (86.89%), exhibit lower accuracy, suggesting that simple majority or WV mechanisms may be less effective in this context. The results highlight the advantage of incorporating F1-scores and confidence-based weighting into ensemble voting. The proposed strategies consistently enhance predictive performance, particularly on datasets with complex decision boundaries and class imbalances.

To evaluate whether the observed performance differences on the synthetic datasets were statistically significant, Wilcoxon signed-rank tests were performed for pairwise comparisons between the proposed voting strategies (ECF1V, CCF1V, and HCF1V) and the conventional voting methods (MV, SV, and WV). The analysis was based on classification accuracies obtained from 10 independent experimental runs using different random seeds for each synthetic dataset. The resulting Wilcoxon test statistics and corresponding p-values are presented in Tables 4, 5 and 6. A significance level of α = 0.05 was adopted throughout the analysis.

**Table 4:** Wilcoxon signed-rank test results—expressed as (Statistic, p-value)—comparing the proposed VSs against existing VSs on Gaussian mixture Dataset.

Voting Strategies	MV	SV	WV
**ECF1V**	(0, 0.002)	(0, 0.002)	(0, 0.002)
**CCF1V**	(0, 0.002)	(0, 0.002)	(0, 0.002)
**HCF1V**	(9, 0.0625)	(6, 0.0273)	(6, 0.0273)

Table 4 summarizes the results of the Wilcoxon signed-rank tests conducted on the Gaussian Mixture Dataset. Both ECF1V and CCF1V achieved statistically significant improvements over all conventional voting methods (MV, SV, and WV), with p-values of 0.002 in all pairwise comparisons. These results provide strong statistical evidence that incorporating class-specific F1-score information enhances ensemble decision-making on this dataset.

HCF1V also demonstrated statistically significant improvements over SV and WV (*p* = 0.0273). However, its improvement over MV was not statistically significant (*p* = 0.0625). This finding suggests that, for the Gaussian Mixture Dataset, selecting only the classifier with the highest class-specific F1-score may not consistently provide sufficient additional benefit over majority voting. In contrast, the cumulative and confidence-enhanced aggregation mechanisms employed by CCF1V and ECF1V appear to offer greater robustness and more consistent performance gains.

**Fig. 13:**
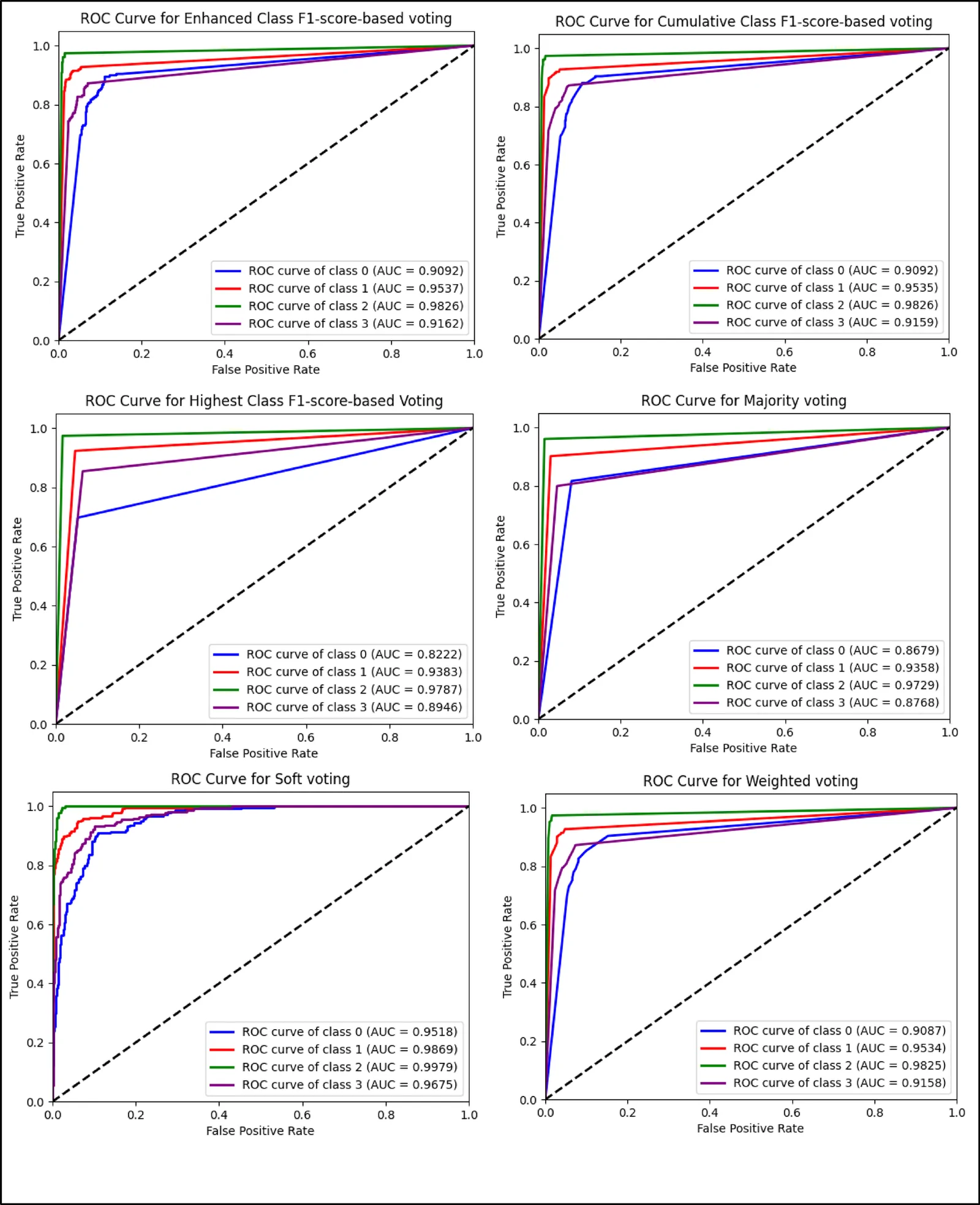
Voting Strategies ROC curves on gaussian mixture dataset.

Figure 13 presents the ROC curves for different VSs applied to the Gaussian Mixture Dataset, comparing both common and proposed ensemble voting methods. Each subfigure illustrates the classification performance of a specific voting strategy, displaying ROC curves for four different classes along with their corresponding AUC values. A higher AUC indicates superior discriminative ability in distinguishing between positive and negative instances.

Among the proposed voting strategies, ECF1V demonstrates the highest classification performance, with AUC scores ranging from 0.9092 to 0.9826, particularly excelling in class 2 (AUC = 0.9826). The steep curves approaching the top-left corner indicate an optimal balance between sensitivity and specificity. Similarly, CCF1V achieves comparably high AUC values, slightly lower than ECF1V for class 3 but maintaining strong performance across all classes. In contrast, HCF1V exhibits lower AUC values, especially for class 0 (AUC = 0.8222), suggesting that relying on a single classifier per class may reduce overall robustness.

For the common voting strategies, MV shows the lowest overall AUC scores, particularly struggling with class imbalance as reflected in its lower performance for class 0 (AUC = 0.8679) and class 3 (AUC = 0.8768). This indicates that simple vote aggregation without weighting may be insufficient for optimal classification in complex datasets. WV slightly improves upon MV, yielding AUC values between 0.9087 and 0.9825, yet still underperforming compared to the proposed F1-score-based methods. SV, on the other hand, achieves the highest AUC for class 2 (0.9979), outperforming all other strategies, and demonstrates exceptional performance across all classes with AUC values ranging from 0.9518 to 0.9979. The results suggest that probabilistic confidence-based aggregation significantly enhances classification accuracy.

The steep initial rise of the ECF1V and SV curves toward the upper-left corner of the ROC space reflects a favorable trade-off between sensitivity and specificity, indicating that both strategies achieve high true positive rates at relatively low false positive rates — a property particularly desirable in multi-class medical classification settings.

Comparing all strategies, ECF1V and SV emerge as the most effective methods, yielding the highest AUC scores and demonstrating superior classification performance on the Gaussian Mixture Dataset. The proposed F1S-VS, particularly ECF1V and CCF1V, consistently outperform traditional MV and WV, highlighting the benefits of incorporating classifier performance metrics for decision-making. HCF1V, while effective, shows lower robustness compared to other proposed strategies, likely due to its reliance on a single classifier per class.

On balanced or near-balanced datasets, per-class F1-scores from well-trained classifiers tend to be relatively uniform across classes, and the proposed weighting scheme naturally converges toward behavior approximating standard Soft Voting. The benefit of F1-score-based class weighting is therefore most pronounced in the presence of meaningful class imbalance, where classifiers exhibit asymmetric per-class performance. Nevertheless, the proposed strategies remain beneficial even under low-imbalance conditions, as any residual variation in per-class classifier competence — however modest — is still captured and reflected in the weight assignment, whereas standard MV and WV remain entirely insensitive to such class-level differences. This is consistent with the results observed on the Gaussian Mixture Dataset, where ECF1V and CCF1V still outperform MV and WV despite the relatively balanced class distribution, confirming that the framework does not require severe imbalance to provide a measurable improvement over traditional voting methods.

### Evaluation on spiral dataset

**Fig. 14:**
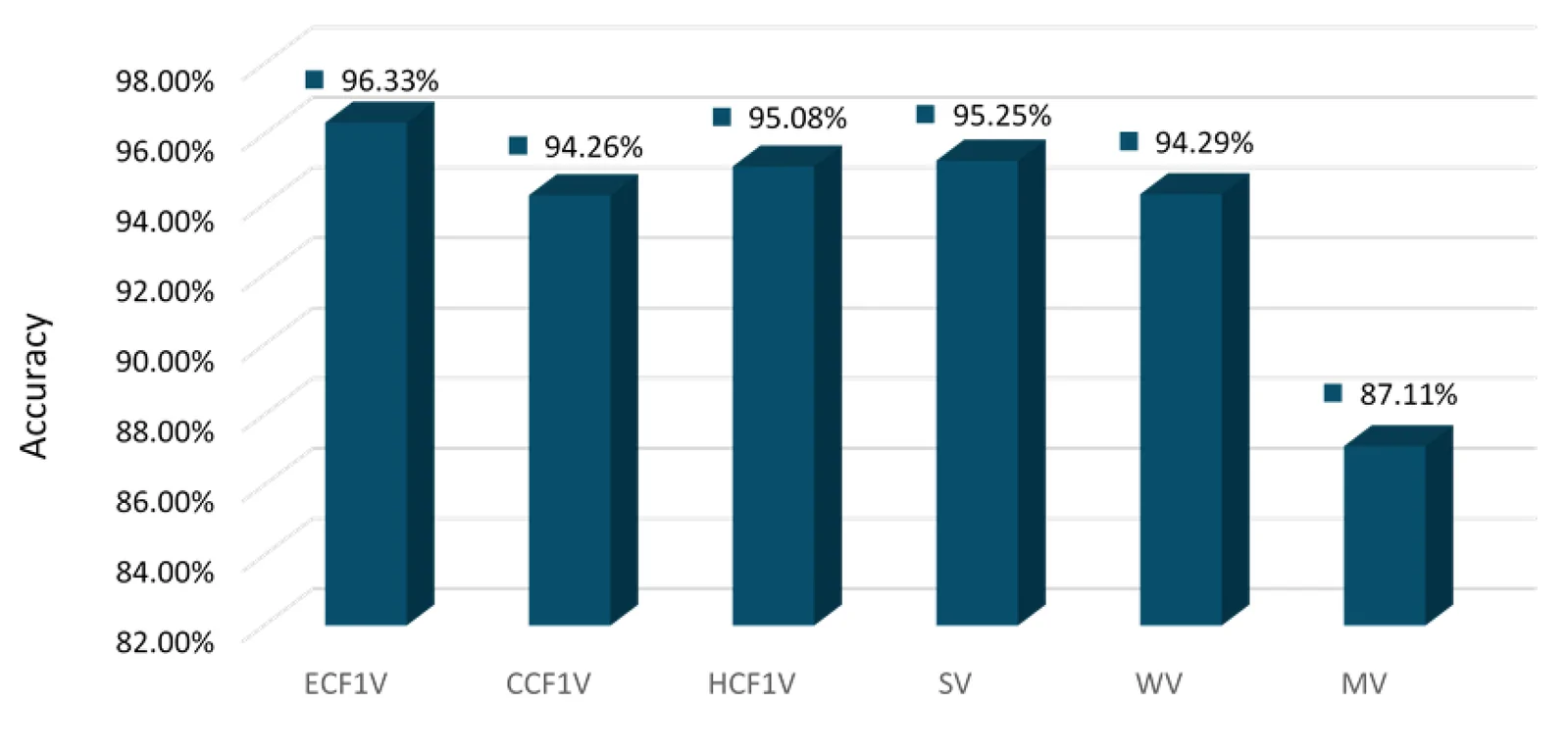
Classification accuracy of six voting strategies evaluated on the Spiral Dataset.

Figure 14 presents the classification accuracies of different VSs applied to the Spiral Dataset, illustrating their effectiveness in handling this complex, non-linearly separable dataset. The bar chart compares the performance of six voting strategies: ECF1V, CCF1V, HCF1V, SV, WV, and MV. Among these methods, ECF1V achieves the highest accuracy at 96.33%, indicating that incorporating both F1-score normalization and confidence-based weighting significantly improves classification performance.

The proposed VSs (ECF1V, CCF1V, and HCF1V) outperform traditional ensemble methods, with CCF1V reaching 94.26% accuracy and HCF1V achieving 95.08% accuracy. These results suggest that prioritizing classifiers based on class-specific F1-scores leads to more reliable predictions, particularly in datasets with overlapping decision boundaries like the Spiral Dataset.

Among the common voting strategies, SV attains an accuracy of 95.25%, demonstrating its effectiveness in probabilistic aggregation, while WV follows closely at 94.29%. In contrast, MV records the lowest accuracy at 87.11%, highlighting its limitation in handling complex datasets where equal-weighted votes may lead to suboptimal decisions.

**Table 5:** Wilcoxon signed-rank test results—expressed as (Statistic, p-value)—comparing the proposed VSs against existing VSs on Spiral Dataset.

Voting Strategies	MV	SV	WV
ECF1V	(0, 0.002)	(0, 0.002)	(0, 0.002)
CCF1V	(0, 0.002)	(0, 0.002)	(0, 1.000)
HCF1V	(0, 0.002)	(1, 0.0059)	(0, 0.002)

Table 5 presents the Wilcoxon signed-rank test results for the Spiral Dataset. Both ECF1V and HCF1V achieved statistically significant improvements over all three conventional voting methods (MV, SV, and WV), with p-values ≤ 0.0059. These results provide strong evidence that the proposed voting strategies offer more effective ensemble decisions on this challenging non-linear dataset.

CCF1V also showed statistically significant improvements over MV and SV. However, no statistically significant difference was observed between CCF1V and WV (*p* = 1.000). This result indicates that the two methods exhibited highly similar performance across the repeated experimental runs. Although CCF1V achieved a slightly higher mean accuracy, the observed difference was not sufficiently consistent to reach statistical significance at the α = 0.05 level.

**Fig. 15:**
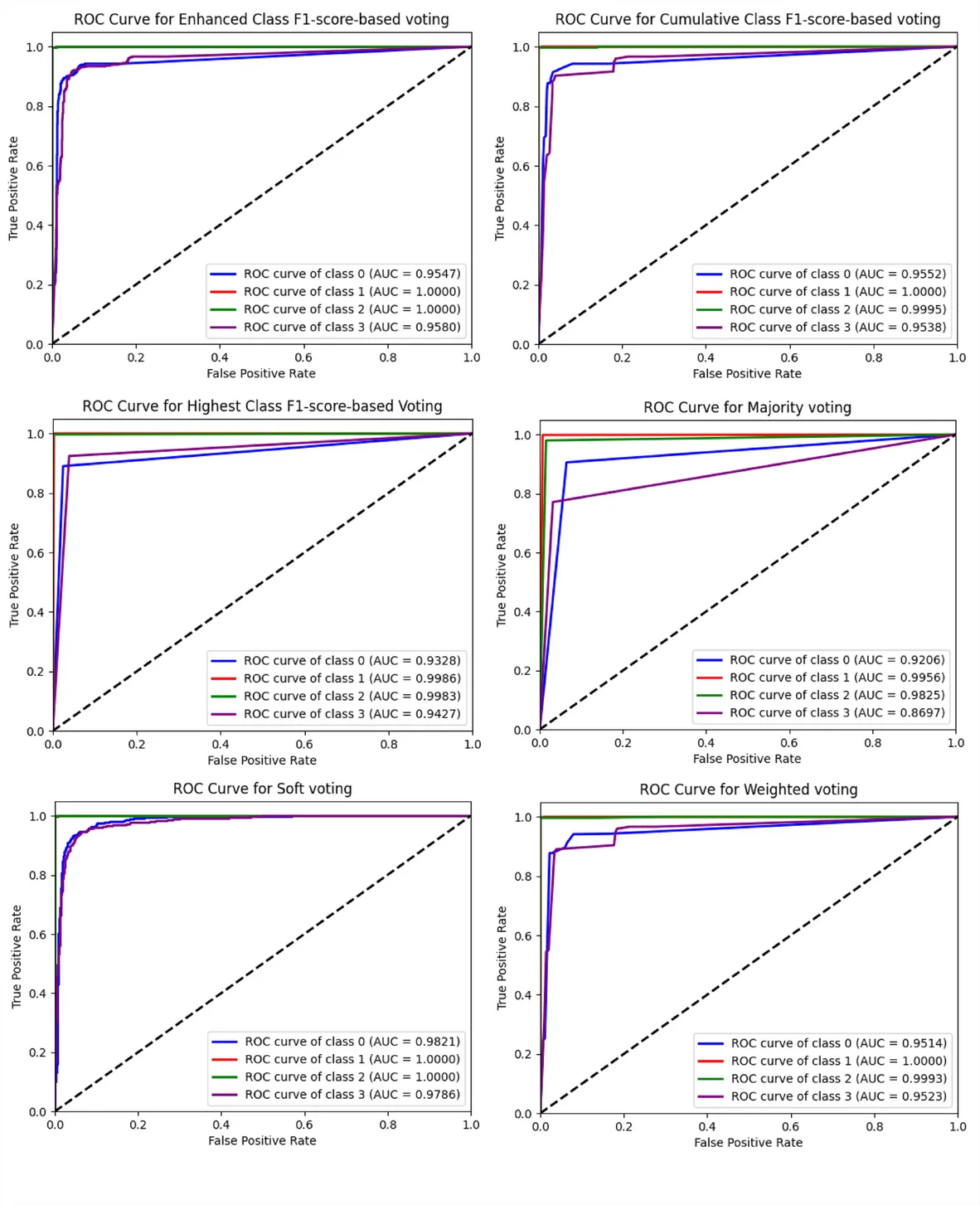
Voting Strategies ROC Curves on Spiral Dataset.

Figure 15 presents ROC curves for various VSs applied to the Spiral Dataset, highlighting their ability to classify different classes effectively. Each subfigure displays ROC curves for four classes, along with their corresponding AUC values, which provide a measure of the discriminative power of each voting strategy.

Among the common voting strategies, SV achieves the highest AUC values across all classes, with AUC scores reaching 1.0000 for classes 1 and 2, and 0.9786 for class 3, indicating superior classification performance. WV also performs well, with an AUC of 1.0000 for class 1, 0.9993 for class 2, and 0.9523 for class 3, demonstrating its ability to incorporate classifier reliability into decision-making. MV, on the other hand, records the lowest AUC values, particularly for class 3 (0.8697), suggesting its limitations in handling class overlaps and complex decision boundaries.

The proposed F1S-VS outperform the common voting strategies, particularly ECF1V and CCF1V. ECF1V achieves perfect classification for classes 1 and 2, with AUC values of 1.0000, and exhibits strong performance for class 3 (0.9580) and class 0 (0.9547), demonstrating its ability to incorporate both F1-score normalization and confidence-based weighting. Similarly, CCF1V achieves an AUC of 1.0000 for class 1, 0.9995 for class 2, and 0.9538 for class 3, highlighting its effectiveness in weighting classifiers based on cumulative F1-scores.

Among the proposed strategies, HCF1V performs slightly worse than ECF1V and CCF1V, with an AUC of 0.9328 for class 0, 0.9986 for class 1, and 0.9427 for class 3. This suggests that while HCF1V effectively selects the highest-performing classifier per class, it may lack the robustness of the cumulative and enhanced F1-score-based approaches.

Comparing all strategies, ECF1V, CCF1V, and SV emerge as the most effective methods, achieving near-perfect classification performance, while MV is the weakest performer, struggling with class imbalances and overlapping regions. The results confirm that F1-score-based ensemble methods significantly enhance classification accuracy, making them particularly beneficial for handling complex, non-linearly separable datasets such as the Spiral Dataset.

The near-perfect AUC values achieved by ECF1V and CCF1V for classes 1 and 2 suggest that the per-class F1-score weighting effectively amplifies the contribution of classifiers that are most discriminative for structurally complex class boundaries, rather than relying on uniform aggregation that may dilute strong individual classifier signals.

### Evaluation on moon dataset

**Fig. 16:**
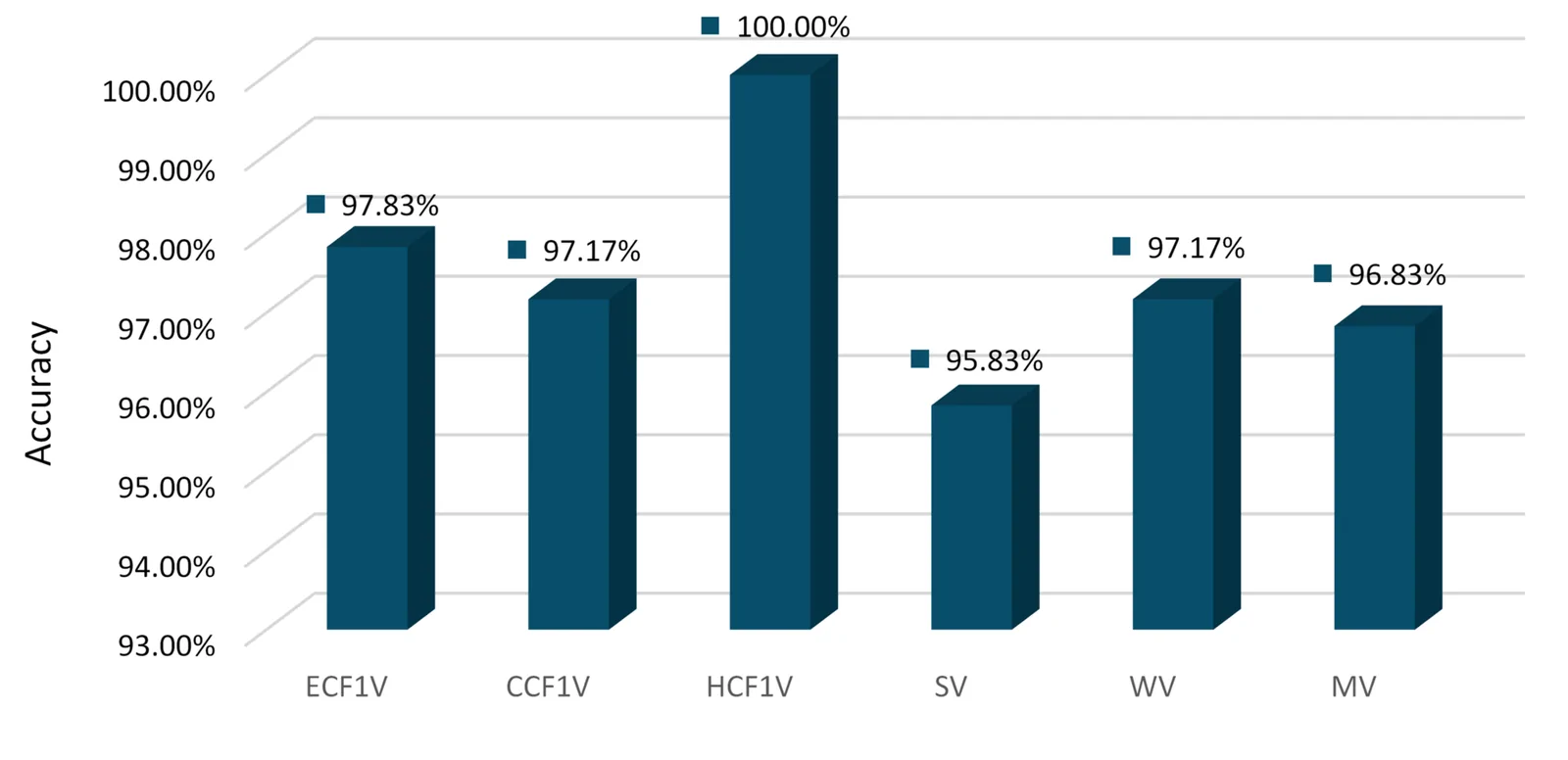
Classification accuracy of six voting strategies evaluated on the Moon Dataset.

Figure 16 presents a bar chart showing the accuracy of various VSs on the Moon Dataset. From the chart, it is clear that the HCF1V method achieves the highest accuracy, at 100%, outperforming all other strategies. This indicates that HCF1V has the most effective approach for this particular dataset, correctly classifying all instances.

Following HCF1V, the ECF1V and CCF1V strategies also perform very well, with accuracies of 97.83% and 97.17%, respectively. These results demonstrate that incorporating F1-scores and class-specific performance metrics significantly improves the ensemble’s predictive accuracy compared to traditional methods.

On the other hand, the SV method achieves an accuracy of 95.83%, which is slightly lower than the F1-based strategies, but still performs well relative to the traditional methods. WV and MV show the lowest performance, with accuracies of 97.17% and 96.83%, respectively. This suggests that simpler, non-weighted or non-probabilistic VSs struggle to handle the complexity of the Moon Dataset, where more sophisticated methods like F1-score-based voting are more beneficial.

Overall, the results highlight the superiority of F1S-VS in handling complex datasets like the Moon Dataset, demonstrating their ability to significantly improve classification accuracy over traditional ensemble methods.

The HCF1V strategy achieves a perfect accuracy of 100% in this case because one of the base classifiers, KNN, has 100% accuracy on the Moon Dataset. In HCF1V, the final class prediction for each test instance is determined by selecting the classifier that has the highest F1-score for each class.

Since KNN is performing at 100% accuracy, it is highly likely that it provides the best predictions for most (if not all) classes in the dataset. In other words, KNN’s predictions are reliable and flawless, and it consistently predicts the correct class for each instance. When applying the HCF1V strategy, because of the high F1-score of KNN, it will dominate the decision-making process, making the final prediction align with KNN’s output. As a result, the HCF1V method benefits from this classifier’s perfect accuracy, leading to a 100% classification accuracy for the ensemble.

The HCF1V strategy does not rely on combining all classifiers’ votes but instead focuses on selecting the most reliable classifier for each class based on its performance (i.e., the F1-score). In this case, KNN stands out as the highest-performing classifier, making it the key contributor to the final decisions, ensuring that HCF1V matches the performance of KNN and achieves 100% accuracy.

This perfect performance illustrates how HCF1V can excel when it dynamically selects high-performing classifiers for each class, particularly when one classifier, like KNN in this case, is perfectly accurate and consistently predicts the correct class.

**Table 6:** Wilcoxon signed-rank test results—expressed as (Statistic, p-value)—comparing the proposed VSs against existing VSs on Moon Dataset.

Voting Strategies	MV	SV	WV
**ECF1V**	(0, 0.002)	(0, 0.002)	(1, 0.00117)
**CCF1V**	(0, 0.002)	(0, 0.002)	(0, 1.000)
**HCF1V**	(0, 0.002)	(0, 0.002)	(0, 0.002)

Table 6 summarizes the Wilcoxon signed-rank test results for the Four Moons Dataset. Both ECF1V and HCF1V achieved statistically significant improvements over all three conventional voting methods (MV, SV, and WV), with p-values ≤ 0.00117. These findings provide strong statistical evidence that the proposed voting strategies consistently outperform traditional ensemble voting approaches on this non-linear multi-class classification problem.

Similar to the results observed for the Spiral Dataset, the improvement achieved by CCF1V over WV did not reach statistical significance (*p* = 1.000). This outcome is consistent with the closely comparable accuracy values reported for these two methods in Fig. 16, indicating that their performance remained nearly indistinguishable across the repeated experimental runs. Although CCF1V achieved a marginally higher average accuracy, the observed difference was not sufficiently consistent to be considered statistically significant at the α = 0.05 significance level.

**Fig. 17:**
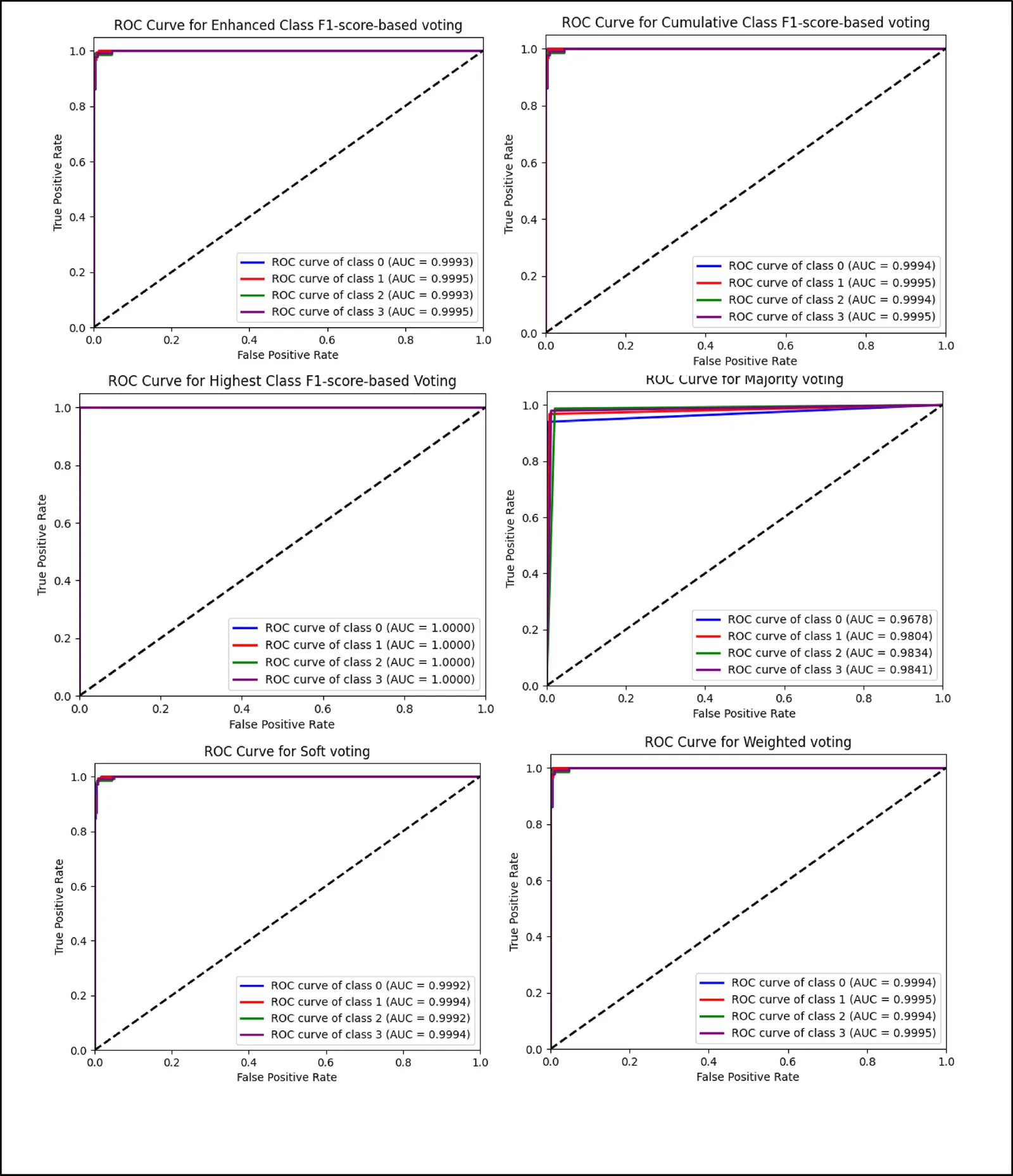
Voting Strategies ROC Curves on Moon Dataset.

Figure 17 presents The ROC curves and corresponding AUC values for various VSs demonstrate notable differences in classification performance. The ECF1V and CCF1V methods perform exceptionally well, achieving AUC values near 1.0000 for all classes. These strategies consistently outperform all other methods, showcasing their robustness, particularly in handling class imbalances and improving class-specific accuracy. Both methods effectively prioritize classifiers that excel in predicting specific classes, leading to nearly perfect predictions.

In comparison, HCF1V also achieves perfect AUC values of 1.0000 for all classes, but it relies on selecting a single classifier with the highest F1-score for each class. While this strategy works well when a classifier is highly accurate across all classes, it may lack flexibility if the chosen classifier is weak for certain instances, making it less adaptable to diverse scenarios.

The SV and WV strategies show high AUC values (around 0.9994), indicating good performance but still falling short of the F1-based methods. SV, which averages the predicted probabilities across classifiers, and WV, which adjusts based on classifier performance, are both effective but may struggle with poorly calibrated classifiers or bias toward dominant models. These methods do not fully capitalize on the performance of each individual classifier, reducing their overall reliability compared to F1-score-based strategies.

MV, on the other hand, shows the weakest performance, with AUC values significantly lower, particularly for class 0 (0.9678). This indicates that MV struggles in complex datasets and scenarios where classifiers have varying levels of performance. Unlike the F1-score-based methods, MV does not consider the reliability or class-specific strengths of individual classifiers, leading to less accurate predictions, especially in imbalanced or challenging classification tasks.

In summary, the F1S-VS (ECF1V and CCF1V) consistently outperform traditional methods like SV, WV, and MV. These F1-based methods provide a more reliable and robust solution, particularly in handling class imbalances and ensuring high classification accuracy in complex datasets.

Across all three synthetic datasets, ECF1V consistently demonstrated statistically significant improvements over MV, SV, and WV (Tables 4, 5 and 6), indicating that its superior performance is robust and unlikely to be attributable to random variation. CCF1V and HCF1V also achieved statistically significant improvements in the majority of pairwise comparisons. The few non-significant results occurred primarily when compared with WV, which generally represented the strongest conventional baseline across the synthetic datasets.

For the real-world datasets (BCWD and UHDD), performance comparisons were conducted against previously published studies. Since these studies report only aggregate performance measures and do not provide access to run-level results or performance distributions, formal statistical significance testing could not be performed. Consequently, the reported comparisons should be interpreted as descriptive rather than inferential. This limitation is inherent to cross-study benchmarking and is acknowledged as a threat to validity in the interpretation of the results.

### Evaluation on BCWD

The base classifiers used for the BCWD dataset were KNN, NB, and DT. These classifiers were selected empirically as the best-performing combination. The same classifier set was used across all six voting strategies (HCF1V, CCF1V, ECF1V, MV, WV, and SV). This ensured a fair comparison. Therefore, any performance differences can be attributed to the voting strategy rather than the choice of base classifiers.

**Fig. 18:**
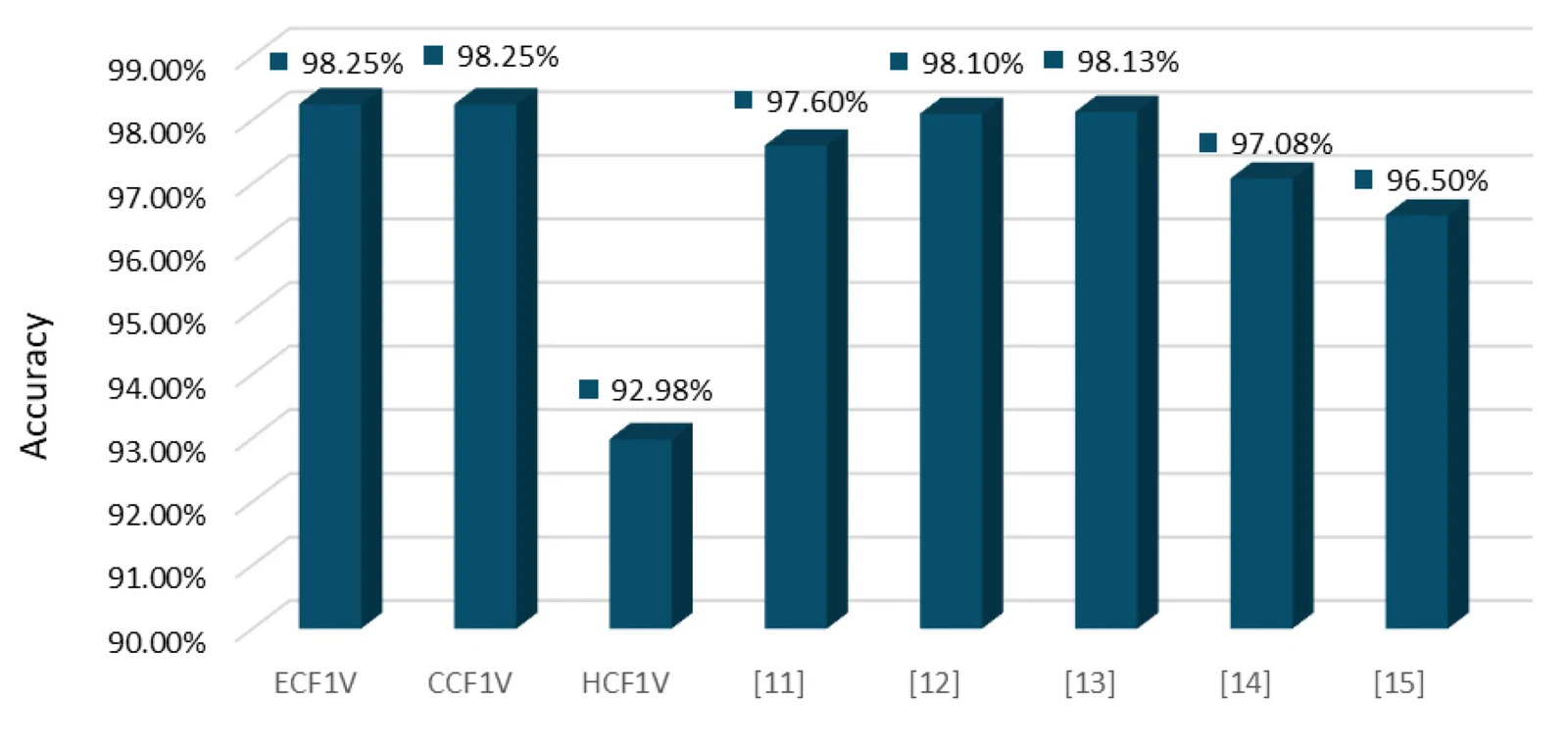
Classification accuracy of proposed VSs and recent studies on the BCWD Dataset.

The bar chart in Fig. 18 presents a comparative analysis of the proposed voting strategies—ECF1V, CCF1V, and HCF1V—against recent studies on the BCWD. The results indicate that ECF1V and CCF1V achieved the highest classification accuracy of 98.25%, outperforming all previously reported methods. Notably, these two strategies surpassed the highest accuracy recorded in prior studies, specifically 98.13% from^14^, confirming their effectiveness in improving predictive performance.

A closer comparison with recent studies reveals that study^14^ attained 98.13% accuracy, making it the closest competitor to the proposed methods. Similarly, study^13^ reported an accuracy of 98.10%, demonstrating strong performance but still trailing behind ECF1V and CCF1V. Study^12^ achieved 97.60% accuracy, while studies^15^ and^16^ reported 97.08% and 96.50%, respectively. These results highlight the superiority of the proposed voting strategies, which leverage F1-score-based weighting to enhance classification reliability.

Among the proposed methods, HCF1V exhibited the lowest accuracy at 92.98%, suggesting that relying solely on the highest F1-score per class may not always be optimal. While this method is effective in certain scenarios, it appears to be less robust compared to the other proposed strategies, particularly when dealing with complex, imbalanced datasets like BCWD.

### Evaluation on UHDD

The base classifiers used for the UHDD dataset were LR, SVM, and RF. These classifiers were selected empirically as the best-performing combination. The same classifier set was used across all voting strategies. This ensured a fair comparison. Therefore, any performance differences can be attributed to the voting mechanism alone.

**Fig. 19:**
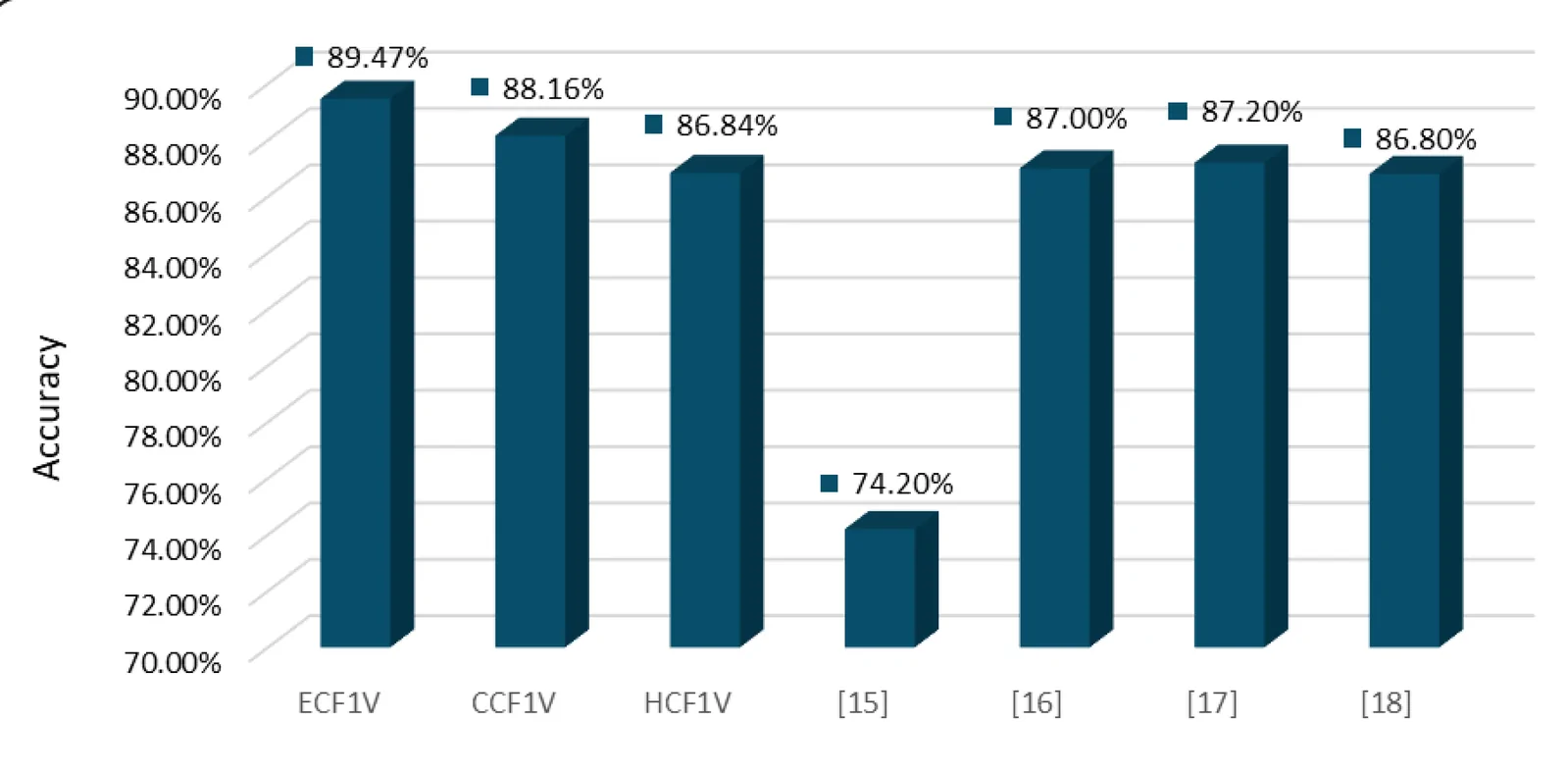
Classification accuracy of proposed VSs and recent studies on the UHDD.

The bar chart in Fig. 19 presents a comparative analysis of the proposed F1S-VS —ECF1V, CCF1V, and HCF1V—against recent studies (^16–18^, and^19^) on the UHDD.

The results indicate that ECF1V achieves the highest accuracy at 89.47%, outperforming all previously reported methods. CCF1V follows closely with 88.16% accuracy, demonstrating the effectiveness of summing F1-scores to enhance classification reliability. HCF1V records an accuracy of 86.84%, confirming its capability in selecting the best-performing classifier per class but highlighting its limitations in overall robustness compared to cumulative and enhanced voting strategies.

When compared to recent studies, studies^17^ and^18^ reported similar accuracies of 87.00% and 87.20%, respectively, which are competitive but still lower than the proposed ECF1V and CCF1V strategies. Study^19^ achieved 86.80% accuracy, slightly surpassing HCF1V but remaining below the best-performing proposed strategies. In contrast, study^16^ exhibited the lowest accuracy at 74.20%, reinforcing the importance of advanced voting techniques for improving classification performance in medical datasets.

A recognized limitation of cross-study comparisons is the heterogeneity of experimental settings, including differences in train-test splits, preprocessing pipelines, and base classifier configurations. The comparisons presented in Figs. 18 and 19 should therefore be interpreted as indicative rather than definitive. Within the controlled experimental setting adopted in this study, the proposed strategies consistently outperform the baseline voting methods under identical conditions, which constitutes the primary basis for our performance claims.

In summary, the empirical evaluations conducted on both synthetic topologies and real-world clinical datasets demonstrate the effectiveness of class-specific dynamic voting frameworks. Traditional ensemble methods, including MV and WV, consistently exhibited lower performance, particularly in problems characterized by complex decision boundaries, significant class overlap, or imbalanced class distributions. In contrast, the proposed F1S-VS achieved notable improvements in classification performance. For highly non-linear datasets such as Spiral, the ECF1V approach, which combines normalized class-specific performance measures with real-time classifier confidence, delivered the highest accuracy, followed by HCF1V and SV.

The advantages of the proposed framework were further validated in healthcare-related classification tasks. On the BCWD, both ECF1V and CCF1V achieved performance comparable to or exceeding that reported by recent state-of-the-art approaches. Similarly, on the UHDD, the proposed methods outperformed conventional ensemble techniques as well as several recent related studies. By incorporating class-specific reliability and instance-level confidence into the voting process, the framework reduces the likelihood of minority-class suppression and improves decision robustness.

Overall, the findings highlight the potential of adaptive class-aware voting mechanisms as an effective ensemble strategy for complex classification problems. Their ability to enhance predictive accuracy while maintaining sensitivity across classes makes them particularly suitable for healthcare decision-support systems, where reliable predictions are essential for informed clinical decisions and improved patient outcomes.

## THreat to validity

Several threats to the validity of the reported findings should be acknowledged. Internally, the per-class F1-scores used to assign classifier weights are computed on a fixed validation split. For minority classes with very few validation instances, these scores may exhibit higher variance across splits, potentially affecting the stability of weight assignments. This concern is most relevant in severely imbalanced settings where minority class representation in the validation set is sparse.

Externally, the proposed strategies have been evaluated on five datasets — three synthetic and two real-world medical benchmarks of moderate size. While these cover a meaningful range of classification scenarios, generalization to larger, high-dimensional datasets with severe imbalance, noisy labels, or missing values cannot be assumed without further empirical validation. Future work will address this by evaluating the proposed strategies on a broader range of clinical and real-world datasets.

## Conclusion

Ensemble learning methods, particularly voting-based strategies, have proven to be effective in improving classification accuracy by leveraging the strengths of multiple classifiers. However, traditional MV, WV, and SV methods often fail to handle non-linear, multi-class, and imbalanced datasets effectively due to their static weighting mechanisms. This limitation is particularly evident in medical classification tasks such as breast cancer and heart disease diagnosis, where misclassification can have severe consequences.

To address these challenges, a dynamic VS is proposed that assigns adaptive class-specific weights to classifiers based on their F1-score performance per class. Three new voting approaches were introduced: HCF1V, CCF1V, and ECF1V. These methods dynamically adjust classifier contributions based on class-specific reliability, ensuring that stronger predictions have greater influence on the final decision.

Experimental evaluation across five datasets demonstrated consistent performance improvements for the proposed strategies over conventional voting methods, with ECF1V achieving the highest accuracy on both real-world medical datasets. These results confirm that per-class F1-score weighting enhances ensemble reliability, particularly under class imbalance conditions.

With respect to **RQ1**, the experimental results obtained across the five evaluated datasets, together with the Wilcoxon signed-rank significance analysis conducted on the synthetic datasets, indicate that class-specific F1-score-based voting strategies generally outperform conventional voting methods (MV, WV, and SV). In most pairwise comparisons, the observed improvements were found to be statistically significant, providing evidence that incorporating class-specific predictive performance into the voting process can enhance ensemble classification effectiveness.

With respect to **RQ2**, ECF1V demonstrated the most consistent performance across datasets exhibiting different levels of class imbalance and decision boundary complexity. While HCF1V achieved the highest performance on certain datasets, particularly the Four Moons Dataset, its performance was less stable across all evaluation scenarios and was surpassed by both CCF1V and ECF1V on the BCWD dataset. Overall, the results suggest that the confidence-enhanced aggregation mechanism employed by ECF1V provides a more reliable balance between accuracy and consistency across diverse classification tasks.

### Key findings

- ECF1V consistently outperforms traditional ensemble methods, providing superior classification accuracy across multiple datasets.
- CCF1V demonstrates strong performance, particularly in handling class imbalances, by aggregating class-specific F1-scores.
- HCF1V, while effective, is less robust than CCF1V and ECF1V, as it relies on a single classifier per class, making it more susceptible to individual classifier weaknesses.
- Traditional MV and WV struggle with complex datasets, while SV performs better but still lacks dynamic adaptability.

### Limitations and future work

While the proposed VSs achieve notable improvements in classification accuracy, there are areas for further enhancement. The computational complexity of ECF1V, due to its reliance on per-class confidence weighting, may need optimization for large-scale datasets. Additionally, expanding the approach to deep learning models and exploring its effectiveness in other domains, such as fraud detection and real-time decision-making, would provide further insights into its adaptability.

The proposed strategies were evaluated on two real-world medical datasets of moderate size and dimensionality. Although the class-specific F1-score weighting mechanism was designed to better capture variations in class-level predictive performance, its effectiveness ultimately depends on the reliability of the underlying base classifiers and the quality of the validation data used to estimate per-class F1-scores. In particular, noisy or unrepresentative validation samples may affect the accuracy of the estimated class weights and, consequently, the final voting decisions. Furthermore, the proposed voting framework operates independently of the feature space dimensionality; therefore, any performance degradation observed in high-dimensional settings would primarily stem from the limitations of the constituent classifiers rather than the voting mechanism itself. Since the current evaluation was conducted on datasets of moderate size and dimensionality, further investigation on larger-scale, high-dimensional, and noisier real-world datasets is needed to assess the generalizability and robustness of the proposed framework.

Future research could also focus on integrating meta-learning techniques to further optimize classifier selection and weight assignment dynamically.

In applications where false negatives are more costly than false positives, such as cancer screening and early disease detection, the F1-score can be replaced with the Fβ-score (β > 1). This gives greater weight to recall. No structural changes to the proposed strategies are required. This represents a practical direction for future work.

## Data Availability

The data that supports the findings of this study are publicly available at [Breast Cancer Wisconsin (Diagnostic) and Heart Disease] and can be accessed via the following link: [ [https://doi.org/10.24432/C5DW2B](https:/doi.org/10.24432/C5DW2B) and [https://doi.org/10.24432/C52P4X](https:/doi.org/10.24432/C52P4X) ].Code AvailabilityThe code implementing the proposed voting strategies (HCF1V, CCF1V, and ECF1V), along with the experimental pipeline used to generate the reported results, is publicly available at [https://doi.org/10.5281/zenodo.21286397].

## Code availability

The code implementing the three proposed voting strategies (HCF1V, CCF1V, and ECF1V), the synthetic dataset generation scripts, and the full experimental pipeline used to produce the reported results are publicly available at [10.5281/zenodo.21895292].
